# Revealing the mystery of metabolic adaptations using a genome scale model of *Leishmania infantum*

**DOI:** 10.1038/s41598-017-10743-x

**Published:** 2017-08-31

**Authors:** Abhishek Subramanian, Ram Rup Sarkar

**Affiliations:** 1 Chemical Engineering and Process Development, CSIR-National Chemical Laboratory, Pune, Maharashtra India; 2 Academy of Scientific & Innovative Research (AcSIR), CSIR-NCL Campus, Pune, India

## Abstract

Human macrophage phagolysosome and sandfly midgut provide antagonistic ecological niches for *Leishmania* parasites to survive and proliferate. Parasites optimize their metabolism to utilize the available inadequate resources by adapting to those environments. Lately, a number of metabolomics studies have revived the interest to understand metabolic strategies utilized by the *Leishmania* parasite for optimal survival within its hosts. For the first time, we propose a reconstructed genome-scale metabolic model for *Leishmania infantum* JPCM5, the analyses of which not only captures observations reported by metabolomics studies in other *Leishmania* species but also divulges novel features of the *L. infantum* metabolome. Our results indicate that *Leishmania* metabolism is organized in such a way that the parasite can select appropriate alternatives to compensate for limited external substrates. A dynamic non-essential amino acid motif exists within the network that promotes a restricted redistribution of resources to yield required essential metabolites. Further, subcellular compartments regulate this metabolic re-routing by reinforcing the physiological coupling of specific reactions. This unique metabolic organization is robust against accidental errors and provides a wide array of choices for the parasite to achieve optimal survival.

## Introduction

Leishmaniasis is a complex, widespread neglected tropical disease in humans caused by *Leishmania* protozoan parasites, spread through infected female sandfly bites. There are around 1 million cases reported for cutaneous leishmaniasis in the last 5 years and between 200,000 to 400,000 infected cases reported annually for the deadlier clinical manifestation, visceral leishmaniasis^1^. The long-standing association with hosts has equipped the parasite with unique metabolic features that support their existence within the hosts^2^. Mechanisms of metabolic adaptation of the *Leishmania* parasite to a variety of external metabolite sources and their stage-specific utilization has been a recurring question^3, 4^. Recent^13^C-isotope-labelled tracing studies in *L. mexicana* indicate that glucose and amino acids, like aspartate, alanine, proline and glutamate, are catabolized through common metabolic routes employed by both the promastigote and amastigote stages of the parasite and propose stage-specific flux changes of pathways that are hard-wired to the differentiation signals within the parasite^5, 6^. The common metabolic routes specify the use of a core set of enzymes within *L. mexicana* metabolism that can metabolize different sources in both stages. The knowledge of these core metabolic routes and enzymes is still incomplete in other *Leishmania* species. Furthermore, the causal reasons for the choice of specific metabolites and their metabolic routes to satisfy the cellular demand of the parasite are still debated upon. One of the speculated reasons is that the differentiation of promastigote to amastigote brings about a lower uptake of non-essential amino acids, higher fatty acid uptake and hypoxic environment in amastigote metabolism as compared to the promastigote stages, that constrains the choice of metabolic routes^5, 6^; although the intrinsic adaptation of the metabolic network structure to the environment is less discussed. Also, the metabolic enzyme repertoire across developmental stages in *Leishmania* (DNA microarrays in *L. major* and mass-spectrometry based quantitative proteomics in *L. infantum*) has been speculated to be constitutively expressed^7, 8^, raising the question of how the flux changes between metabolic states for the two stages might be achieved. This is also supported by the observations from whole genome comparisons of codon usage among different species of *Leishmania*, which outline very few differences within metabolic genes between the species^9^. All the above observations motivated us to investigate the role of the underlying metabolic network structure in metabolic adaptation of *L. infantum* to the host environment.

Genome-scale metabolic models of eukaryotic parasites provide a comprehensive overview of metabolic pathways and attempt to understand the stage-specific nature of parasite metabolism^10–13^. No genome-scale metabolic reconstruction is available for *Leishmania infantum* till date. For the first time, we propose a new manually reconstructed genome-scale metabolic network model (iAS556) for the species *Leishmania infantum* JPCM5 (a well characterized *L. infantum* strain), consisting of 1260 reactions and 1160 metabolites (see Supplementary Data S1, S2, Methods, and Sections 1, 2 of Supplementary Information). We hypothesize that the underlying reaction stoichiometry and reversibility present within the *L. infantum* metabolic network are adequate to explain these specific route selections, which remain conserved across developmental stages; although, the corresponding flux changes are strong-armed by constraints on uptake of environmental metabolites. Auxiliary to the hypothesis, analyses of the proposed *L. infantum* genome-scale constraint-based model reveals the simplicity of metabolome organization within *Leishmania* and its utility to achieve complex metabolic phenotype traits for optimal usage/synthesis of essential metabolites under varying environments. Related to this, the factors that govern metabolome organization and their effects on distribution of metabolites are also unknown. Our results suggest that the coupling of specific reactions for reasons of mass, redox and energy balance, driven by subcellular compartmentalization of enzymes might be the most vital component for appropriate distribution of metabolites within the network. This organization ensures unaltered production of biomass metabolites despite random changes occurring within the parasite metabolome.

## Results

### Formulation of *Leishmania-specific* metabolic demand reaction

A *Leishmania-specific* metabolic demand reaction was formulated using relative signal intensities per cell protein of metabolites normalized to the sum intensity of the total metabolite pool reported for log phase *L. donovani* promastigotes averaged for 3–6 days of *in vitro* growth^14^. In this experiment^14^, an untargeted metabolomics approach was used to obtain detailed information of the metabolite changes occurring within different developmental stages of promastigote under defined media conditions. Further, promastigotes were collected from days 3 to 6 of *in vitro* growth representing the whole procyclic to metacyclic transition of the parasite and was analyzed using liquid chromatography-mass spectrometry (LC-MS). Results from this study were applied to represent the total metabolite pool within the parasite for the given time span of growth. The signal intensity per cell protein of each metabolite averaged for 3–6 days was normalized to total metabolite signal intensity per cell protein (eq. 1). The normalized intensities thus, represents the probable fraction of each metabolite available in the total metabolite pool (expressed as total metabolite signal intensity per cell protein) that can be used for generating unit biomass in the given growth phase.

1 $${\rm{Available}}\,{\rm{fraction}}\,{\rm{of}}\,{\rm{metabolite}}=\frac{{\rm{Signal}}\,{\rm{intensity}}\,{\rm{per}}\,{\rm{cell}}\,{\rm{protein}}\,{\rm{of}}\,{\rm{metabolite}}\,{\rm{in}}\,\mathrm{promastigote}(\mathrm{averaged}\,{\rm{for}}\,3-6\,\mathrm{days})\,}{{\rm{Total}}\,{\rm{metabolite}}\,{\rm{signal}}\,{\rm{intensity}}\,{\rm{per}}\,{\rm{cell}}\,{\rm{protein}}}$$

This available fraction represents the coefficient of that metabolite in the metabolic demand reaction (see Section 3, Supplementary Information for details). Previous genome-scale reconstruction studies in other parasites have used biochemical data from distant organisms for creation of the metabolic demand^11, 12^. As the metabolic demand reaction of *L. infantum* could not be completely established based on available literature, and *L. donovani* being evolutionary related to *L. infantum* ^9^, as a proof of concept, the formulated metabolic demand reaction can be applied to *L. infantum*. Similar usage of metabolomics data for formulation of objective function was also considered in the *L. infantum* energy metabolic network^15^, which gave comparable results with experiments. Similarly, the results in this study indicate that the formulated metabolic demand reaction can capture the essential, realistic distribution of fluxes within the iAS556 metabolic network. Furthermore, a recent study^16^ demonstrates that, between the *L. infantum* and *L. donovani* species, the ratio of membrane phospholipids known to be produced by the parasite (considered within the metabolic demand reaction) [Phosphatidylcholine (PC): Phosphatidylethanolamine (PE): Phosphatidylinositol (PI)] remains similar across the two species in both stages; supporting the use of the metabolic demand reaction curated from *L. donovani* data in simulating the phenotypes of *L. infantum*.

### Prediction of stage-specific metabolic routes for catabolism of major carbon sources

**Figure 1:**
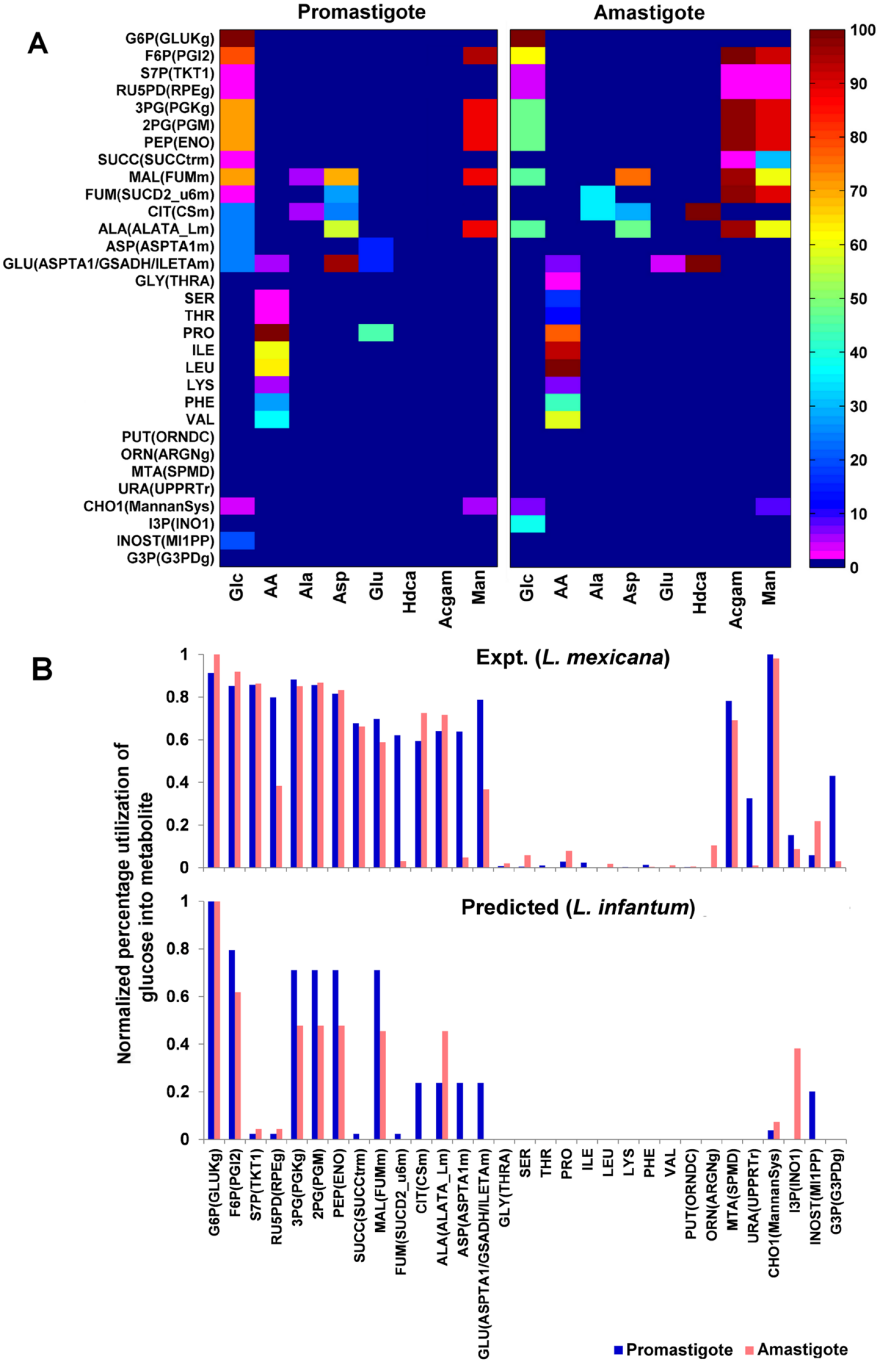
Fate of environmental metabolites within the *L. infantum* iAS556 metabolic network. (**A**) Metabolic utilization of carbon sources in developmental stages of *Leishmania infantum* **–** Each color in the heatmap indicates the percentage utilization of a given input metabolite (X-axis) for formation of a given internal metabolite when optimized for the metabolic demand reaction. The metabolic demand reaction represents a drain (overall net conversion) of precursor metabolites at their relative stoichiometry. In this case, the chosen metabolites represent the metabolites that form biomass. It is also important to note that only metabolites produced by the parasite are included within the metabolic demand reaction. (**B**) Comparison between promastigote and amastigote stages as given in the metabolomics experiment, where ^13^C isotope enrichment of each metabolite was expressed in mole percentage & normalized to glucose-6-phosphate for parasites grown on ^13^C labeled glucose^5^ and reaction fluxes (normalized to hexokinase/glucose uptake) as predicted for the glucose-only situation from the model. Blue colored bars indicate the promastigote metabolic state and red colored indicate the amastigote state. The metabolites in the X-axis and the order in which they are arranged, were taken from the aforementioned ^13^C isotope enrichment experiment performed in *L. mexicana* ^5^. The details of comparisons of flux-profiles between experiment and predicted in both the stages is given in Section 5, Supplementary Information.

Previous studies characterize a low uptake of non-essential amino acids, fatty acid uptake to compensate for the scarcity of glucose, reduced secretion of overflow metabolites, a hypoxic and acidic environment as factors governing the metabolic state within the infective amastigote stage of the parasite^5, 17^. Also, the possible strategic routes to utilize a variety of carbon sources within the two stages, were also demonstrated^5^. Given these boundary (exchange) constraints (Section 4 of Supplementary Information), we asked the question whether the present constraint-based model demonstrates similar metabolic routes in the *L. infantum* network or not. Re-creating the above-mentioned scenarios, the fate of different carbon resources between promastigote and amastigote metabolic states was predicted (see Methods) and compared (Fig. 1A, Supplementary Table S1). The predicted reaction steady state fluxes represent the stoichiometrically adjusted percentage conversion of a given substrate (fate) into a subsequent product. For example, glucose is entirely converted to glucose-6-phosphate (same stoichiometry). Hence, flux of glucokinase is equal to flux through glucose uptake and represents a 100% conversion of total input glucose into glucose-6-phosphate. With sufficient availability (promastigote), glucose was largely driven towards optimal production of overflow metabolites (2.34%), adenosine monophosphate (AMP) formation (2.34%), phospholipids (20.2%), glutamate (23.7%), aspartate (23.7%), alanine (23.7%) and mannan (3.9%); further, characterized by NAD redox coupling occurring between glycolysis and succinate fermentation, entry of pyruvate, succinate and glutamate into the tricarboxylic acid cycle (TCA), and regeneration of NAD reducing equivalents from C4-dicarboxylic acids by oxidative phosphorylation^6^. In the amastigote, which experiences a decrease in glucose uptake, the quantity of glycolytic flux is largely compromised. The preference of glucose utilization is relatively more towards AMP formation (4.41%) and mannan synthesis (7.36%) when compared with the sufficient glucose situation. The remaining glucose is utilized towards alanine formation (45%) and phospholipid formation (38.27%), whereas asparagine, tyrosine and isoleucine are utilized for formation of glutamate, glutamine and proline.

As opposed to glucose, majority of alanine was utilized for satisfying the cellular demand; some of it (6.05%) was converted to pyruvate, which was either utilized for CO_2_ fixation within glycosome or secreted as overflow in both abundant and constrained scenarios. Similarly, environmental aspartate in the promastigote is consumed and transaminated into glutamate (96.83%) and oxaloacetate in cytoplasm^6^. Oxaloacetate forms malate, which enters mitochondria as fumarate, ultimately forming alanine (56.69%). In the amastigote, aspartate uptake reduces despite ﻿being catabolized via a similar route, the only difference being the sole formation of alanine (47.83%). Sufficient environmental glutamate is used for synthesis of glutamine (14.72%) via glycosomal GMP synthase, synthesis of proline (44.74%), aspartate by reverse de-amination of glutamate (14.72%), and glutamylcysteine to form trypanothione. In the amastigote, reduced glutamate uptake coerces its utilization into cellular metabolic demand and not as a precursor. Proline uptake (AA in Fig. 1, see Methods) is preferred as the sole carbon source to produce glutamate, in order to satisfy the metabolic demand in absence of environmental glutamate (5.14%). Serine, lysine, phenylalanine and valine (AA in Fig. 1) neither catabolize to give any intermediates nor are used as precursors and are directly utilized for biomass^5^. Under hypoxic conditions (amastigote condition), as biomass formation is sub-optimal, their utilization into biomass decreases.

Glycine was produced solely from threonine. Leucine and isoleucine, both are utilized for provision of reduced FAD within mitochondrion. In the process, isoleucine converts into glutamate. As glutamate uptake largely reduces in amastigote, formation of glutamate and proline is largely compensated by isoleucine and leucine. Fatty acids are preferred only under glucose deficient conditions (amastigote metabolic state) and primarily provide acetyl-coA to pyruvate dehydrogenase complex^18^, thereby eventually forming glutamate. All the aforementioned predictions have also been experimentally observed from metabolomics studies^5^. In addition to the above carbon sources, the utilization of amino sugars (N-acetyl glucosamine) and mannose was also investigated. N-acetyl glucosamine is exclusively utilized under intracellular glucose-deficient, hypoxic conditions. N-acetyl glucosamine and mannose, both are utilized for provision of fructose-6-phosphate, which enters into mitochondria via succinate formation to produce alanine (98.3% and 87.76%, respectively). The uptake rates of both these sugars were predicted to decrease under hypoxic conditions of the amastigote; although, they do compensate the scarcity of glucose required for production of non-essential amino acids and mannan^4, 19^. In addition, mannose also forms mannan (4.89%) and compensates the reduction in glucose uptake within the amastigote. To further support the accuracy of model predictions, experimentally observed knockout phenotypes were compared with model-predicted phenotypes. 81% known phenotypes in promastigote and 84% known phenotypes in amastigote situations were accurately predicted from the model (Section 6 of Supplementary Information).

The stage-specific comparison between promastigote and amastigote flux-profiles as predicted by the model and^13^C isotope enrichment of metabolites, for the glucose-only situation is given in Fig. 1B. It is important to note that, as there was no such targeted^13^C metabolomics study available for *L. infantum* JPCM5 and because the metabolic microenvironment experienced by different *Leishmania* species is rather similar^4^, the predicted reaction fluxes were compared with the ^13^C isotope enrichment of metabolites reported in the metabolomics study available for *L. mexicana*. Normalization was performed for percentage values of fluxes and experimentally available ^13^C isotope enrichment in a 0 to 1 scale, where 1 represents the highest normalized percentage flux value/isotope enrichment in mole percent and 0 the lowest. From Fig. 1B, it is clear that amastigotes demonstrate a lower synthesis of majority metabolites from glucose as compared to promastigotes owing to the reduced glucose uptake, in both experiments and model. Mannan production during the amastigote stage is larger than the promastigote in our *L. infantum* model, as compared to *L. mexicana*. The upregulation of mannan synthesis in amastigote was also observed in previous studies^4^. A slight upregulation of the pentose-phosphate shunt was observed in the *L. infantum* amastigote indicating its role in satisfying the requirement of AMP under a reduced glucose uptake. It can also be observed that similar metabolites are produced from glucose in both *L. infantum* and *L. mexicana* but with significant quantitative differences in both stages. The quantitative differences observed between the predicted reaction steady state fluxes in *L. infantum* JPCM5 and the^13^C isotope enrichment of metabolites available for *L. mexicana* might be due to – a) species-specific genes unique to *L. mexicana* or *L. infantum*. For example, metabolites of the incomplete urea cycle, are largely independent of glucose utilization and are optimally produced from different carbon sources like arginine for utilization into the metabolic demand within the *L. infantum* network which might not be the case in *L. mexicana*, due to differences in underlying network structure, b) multiple subcellular locations of different enzymes that might lead to alternative paths for distribution of intermediate metabolites produced from glucose within the pathways of glycolysis, pentose-phosphate-pathway and non-essential amino acid synthesis unique to the *L. infantum* JPCM5 metabolic network, and/or, c) metabolic demand being different between *L. infantum* and *L. mexicana*, leads to an increased drain of intermediate metabolites like non-essential amino acids into the *L. infantum* metabolic demand as compared to *L. mexicana*. The aforementioned reasons are only suggestive of the possibilities underlying the observed differences. Further inferences based on comparison of flux profiles in the two species can only be performed from an evolutionary perspective, given the degree of evolutionary divergence between *L. infantum* and *L. mexicana*, and the relatedness of *L. mexicana* with the Sauroleishmania^20^, which can be addressed in a future work.

### Dynamic role of the non-essential amino acid motif in metabolic flux re-organizations

The observation of glucose being primarily diverted towards alanine under glucose-deficient conditions specifies that glucose can be substituted by a variety of other carbon sources to fulfill metabolite requirements other than alanine. The foremost question here is how this substitution can be achieved? To answer this, we introduce the notion of a “non-essential amino acid motif”, which re-routes different metabolic inputs towards specific outputs under absence of preferred carbon sources, via implicit non-essential amino acid inter-conversions. The motif comprises of glutamate dehydrogenase, aspartate aminotransferase, tyrosine catabolic pathways, proline biosynthesis, glutamine biosynthesis, incomplete urea cycle, glycolytic and succinate fermentation pathways. To investigate their functionality, a sensitivity analysis was performed to understand the effect of glucose and uptakes of all amino acids, while optimizing for the formation of glutamate, alanine, aspartate, glutamine, proline, glycine, myoinositol and mannan, the formation of each considered as a separate objective function (see Methods). Apparent optimal production of each amino acid due to self-uptake was not included in analysis. Further, the metabolic demand reaction was constrained to zero flux in each case.

**Figure 2:**
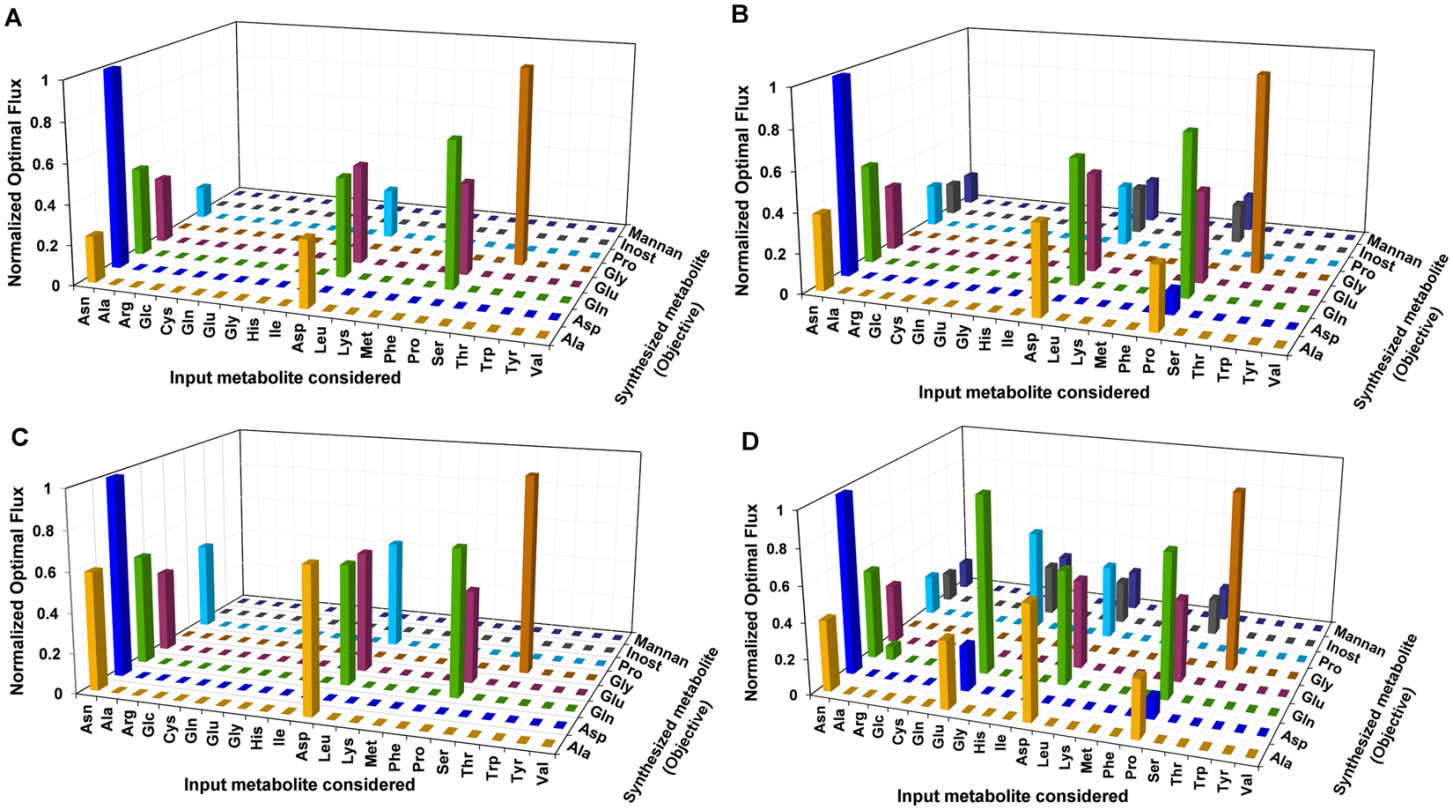
Sensitivity of flux towards synthesis of internal metabolites to specific input metabolite uptakes – Each bar in the plot represents the maximum flux through the synthesis of specific internal metabolites due to specific input metabolites, each synthesis considered as a separate metabolite drain (objective function) in the model. Four different scenarios were considered for optimizing the considered objective functions (synthesis of each separate output metabolite) namely, (**A**) only one given input at a time; (**B**) two inputs given at a time, glucose being fixed and a variable second input; (**C**) two inputs given at a time, one being isoleucine, which is kept fixed and a variable second input; and (**D**) Three inputs given at a time, glucose, tyrosine are fixed and a variable third input. The details of the designed methodology are further explained in Methods section.

Alanine is formed from a variety of resources and hence, tends to be an important overflow metabolite^21, 22^. Asparagine and aspartate, each of them when singly present in the environment can sub-optimally produce alanine (Fig. 2A). Presence of environmental glucose, isoleucine and tyrosine along with asparagine or aspartate amplifies production of alanine. Alanine is also produced when both proline and glucose are present in the environment, or when glutamate, glucose and tyrosine are made available. Glucose and tyrosine provide excess glutamate (Fig. 2B). Aspartate is optimally formed from asparagine, but sub-optimally formed from environmental proline and glucose or glutamate, glucose and tyrosine combinations. Similarly, glutamine is strictly formed when asparagine, aspartate and proline are part of the environment. Aspartate and isoleucine in the environment amplify flux towards production of glutamate, glutamine and proline (Fig. 2C). Glucose and tyrosine increase flux through proline degradation and glutamate formation, thereby producing excess glutamine (Fig. 2D). Glutamate biosynthesis follows the same route as glutamine. Proline, like any other non-essential amino acid is formed primarily from environmental asparagine and aspartate. Glutamate uptake from environment can also supplement glucose and tyrosine to form proline. The choice of various input metabolite combinations to produce a precise repertoire of outputs is largely governed by their ability to generate internal glutamate as a precursor. The importance of glutamate has also been previously discussed in the context of energy metabolism^15^. Also, these metabolite utilizations are typically due to presence of a redox and energy balance between the reactions utilizing these metabolic precursors.

Environmental glucose and phosphatidic acid are catabolized through linear pathways for synthesis of mannan and phospholipids, respectively; although it is dependent on the non-essential amino acid motif via argininosuccinate synthetase for provision of ATP. Model-based knockout of argininosuccinate synthetase also predicts a lethal phenotype suggestive of its essential requirement (Section 6 of Supplementary Information). This further provides a novel insight into the possible essential role of argininosuccinate synthetase within the glycosome^23^.

### Subcellular compartmentalization induces metabolite flux dependencies between distinct reactions

The utility and catabolism of environmental metabolites through defined metabolic routes is largely influenced by coupling between pathways for regaining electrons through reducing equivalents, ATP, small molecules, ions and other cofactors lost by one pathway from another pathway^8, 24^. The underlying network structure and its flux adaptation to the external sources are largely dictated by these dependencies. Intracellular boundaries created by subcellular compartments further reinforce these flux couplings^25, 26^. Glycosome and mitochondrion together harbor around 20% of metabolic enzymes (Section 2 of Supplementary Information) that produce biomass metabolites. Formations of only few metabolites, like sterol and membrane lipids (around 4%), are restricted within the endoplasmic reticulum. Comparing scenarios for the presence and absence of the glycosome and mitochondrion, effect on pairing of reactions based on mass balance was studied. Simulations were performed in both the model-presumed promastigote and amastigote conditions for both the normal (subcellular compartment present) and perturbed (subcellular compartment absent) scenarios (Supplementary Tables S2 and S3).

**Figure 3:**
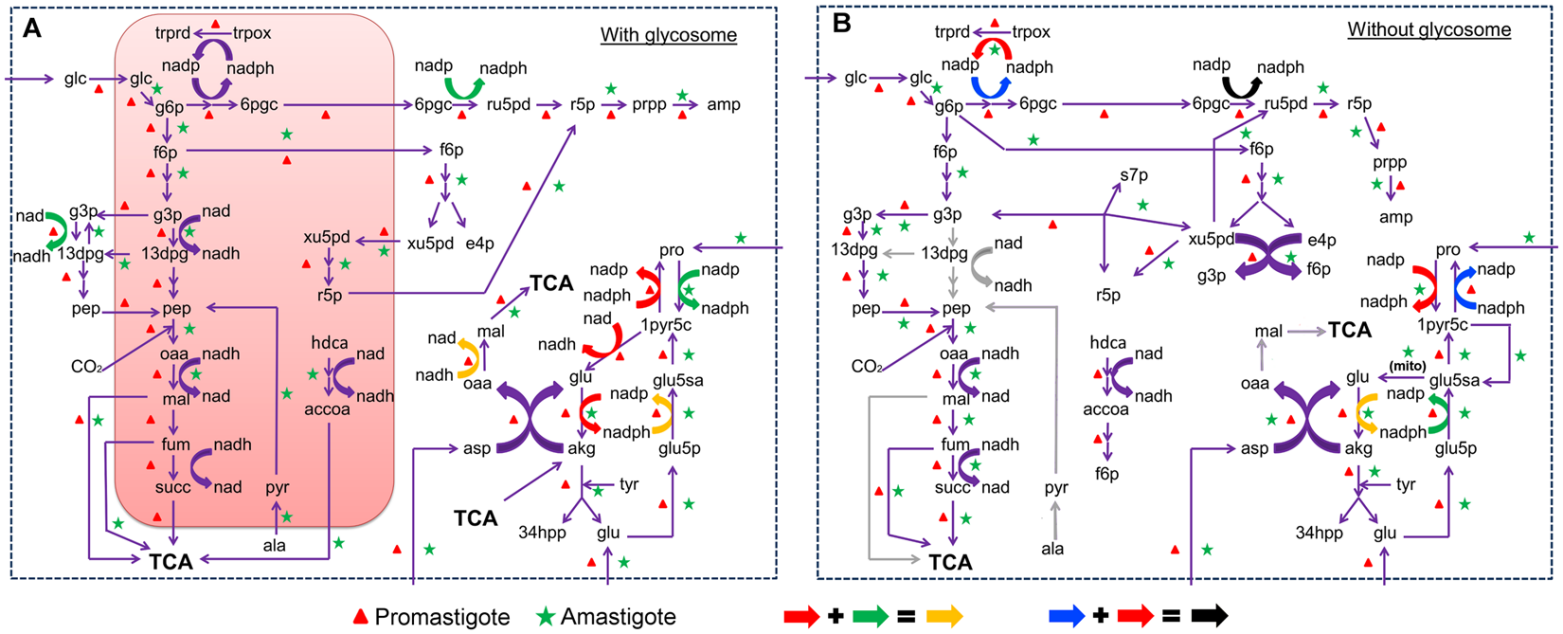
Comparison of flux profiles. (**A**) Normal (with glycosome) scenario. Red colored box indicates the glycosome. (**B**) Perturbed (﻿without glycosome) scenario – the reactions take place in cytoplasm (represented as a bounded white space). While arrows indicate conversions from one metabolite to another, the colors indicate the mass balance of redox equivalents between a set of reactions. Grey colored arrows indicate complete blockage of reactions that were present in the actual normal scenario. The cases where redox balance is maintained within more than one reaction, are indicated by combination of colored arrows, as shown in the bottom of the figure.

Glucose is catabolized for AMP synthesis, diversion into TCA for non-essential amino acid synthesis via succinate fermentation, myo-inositol and mannan formation (Fig. 3A). Given the absence of glycolytic regulation machinery in Trypanosomatids^27^, glycosomes supervise this utilization of glucose towards appropriate outputs by promoting redox coupling between upper and lower part of glycolysis via NAD, glycosomal trypanothione reductase and pentose phosphate shunt via NADP, and cytosolic glutamate dehydrogenase with proline biosynthesis again via NADP, in glucose sufficient conditions. This dictates the use of precise combinations of external metabolites and their directed catabolism towards various internal metabolites via the non-essential amino acid motif. The glycosome also restricts the exchange of ATP/ADP and NAD/NADH generated from glycosomal reactions into the cytoplasm, thus, creating a balance between them^28^. This balance is maintained by upper and lower part of glycolysis, argininosuccinate synthetase, and nucleotide salvage pathways within the glycosome. The above-mentioned reactions are coupled even in deficient glucose conditions (amastigote), although the drain of glucose into succinate fermentation for formation of alanine is largely compromised. This is compensated by the NAD redox coupling between succinate fermentation and glycosomal fatty acid *β*-oxidation, governed by the presence of glycosome. The specificity of fatty acids as preferred carbon sources only in deficient glucose conditions is thus regulated by the glycosome. Removal of glycosome (Fig. 3B) gives rise to a distinct flux profile under glucose-deficient conditions when compared with normal flux scenarios (*P < *0.05); although under glucose-sufficient conditions, the generated profiles seem to be similar (Fig. S3A and B, Section 7 of Supplementary Information).

**Figure 4:**
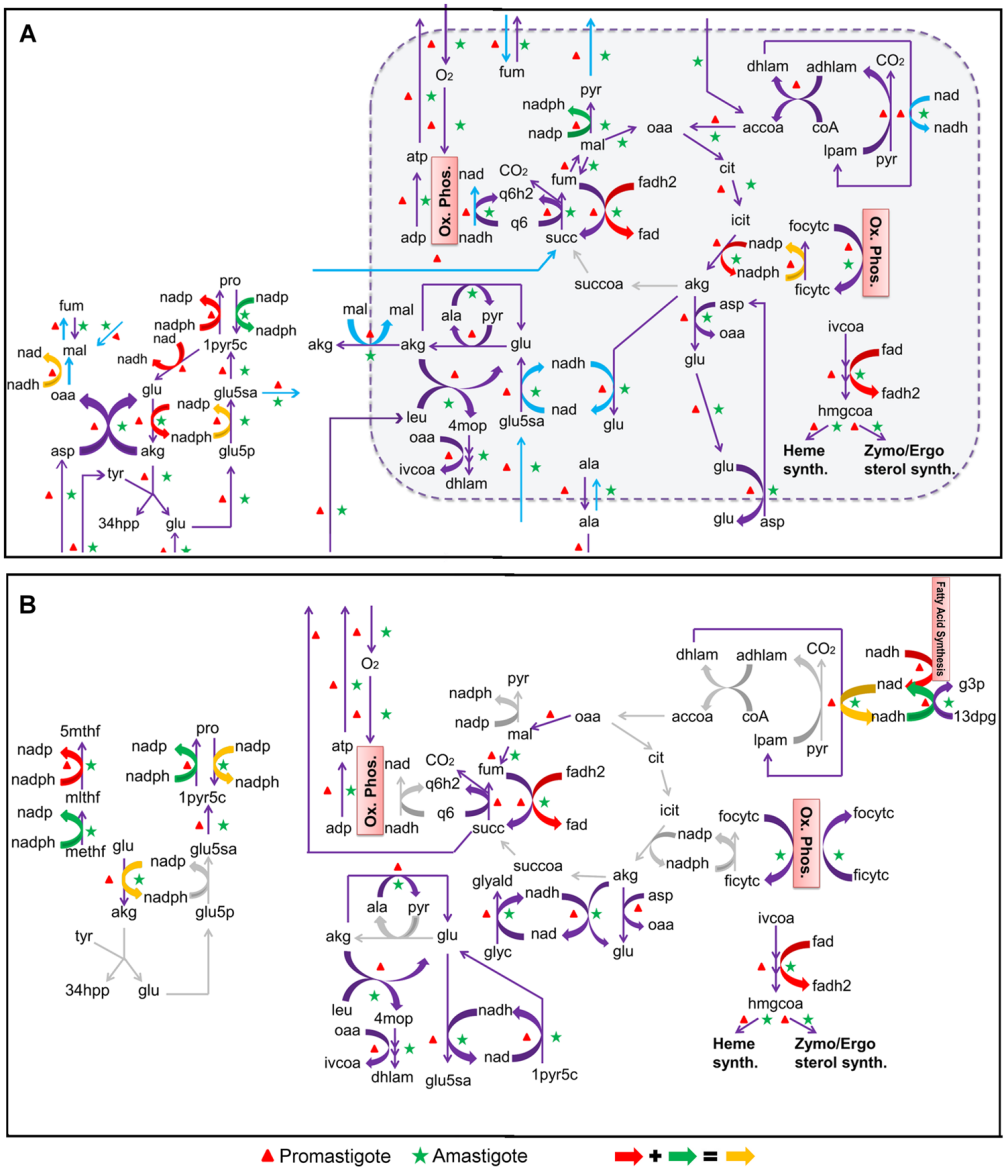
Comparison of flux profiles. (**A**) Normal (with mitochondrion). Blue colored box indicates the mitochondrion. (**B**) Perturbed (﻿without mitochondrion) – the reactions take place in cytoplasm (represented as a bounded white space). While arrows indicate conversions from one metabolite to another, the colors indicate the mass balance of redox equivalents between a set of reactions. Grey colored arrows indicate complete blockage of reactions that were present in the actual normal scenario. The cases where redox balance is maintained within more than one reaction, are indicated by combination of colored arrows, as shown in the bottom of the figure.

A single mitochondrion exists in *Leishmania* that contains enzymes of TCA cycle, oxidative phosphorylation, amino acid catabolism, fatty acid biosynthesis and initial steps of sterol biosynthesis, each of which play an important role in the survival of the parasite^29^. The presence of mitochondrion directs tyrosine and glucose into the non-essential amino acid motif to produce glutamate, glutamine, proline, aspartate and alanine. One of the most important functions of the mitochondrion is to redirect C4-dicarboxylic acids produced from glycolysis and non-essential amino acid motif to synthesize mitochondrial glutamate, thereby driving the TCA to produce reducing equivalents for oxidative phosphorylation (Fig. 4A). The mitochondrion also ensures utilization of tyrosine catabolism to provide excess glutamate for proline biosynthesis, protection of alanine for meeting cellular demand and restricts the excess drain of useful succinate into the environment to provide adequate FAD reducing equivalents for initial steps of sterol synthesis. Similar to the glycosome, removal of mitochondrion (Fig. 4B) gives rise to a distinct flux profile under glucose-deficient conditions when compared with normal flux scenarios (*P* < 0.05); although under glucose-sufficient conditions, the generated profiles seem to be similar (Fig. S3C and D, Section 7 of Supplementary Information). The comparisons of metabolite utilizations for the normal and both the perturbed scenarios have been discussed in Supplementary Information.

### Physiological coupling between metabolic reactions is robust against random reaction deletions

**Figure 5:**
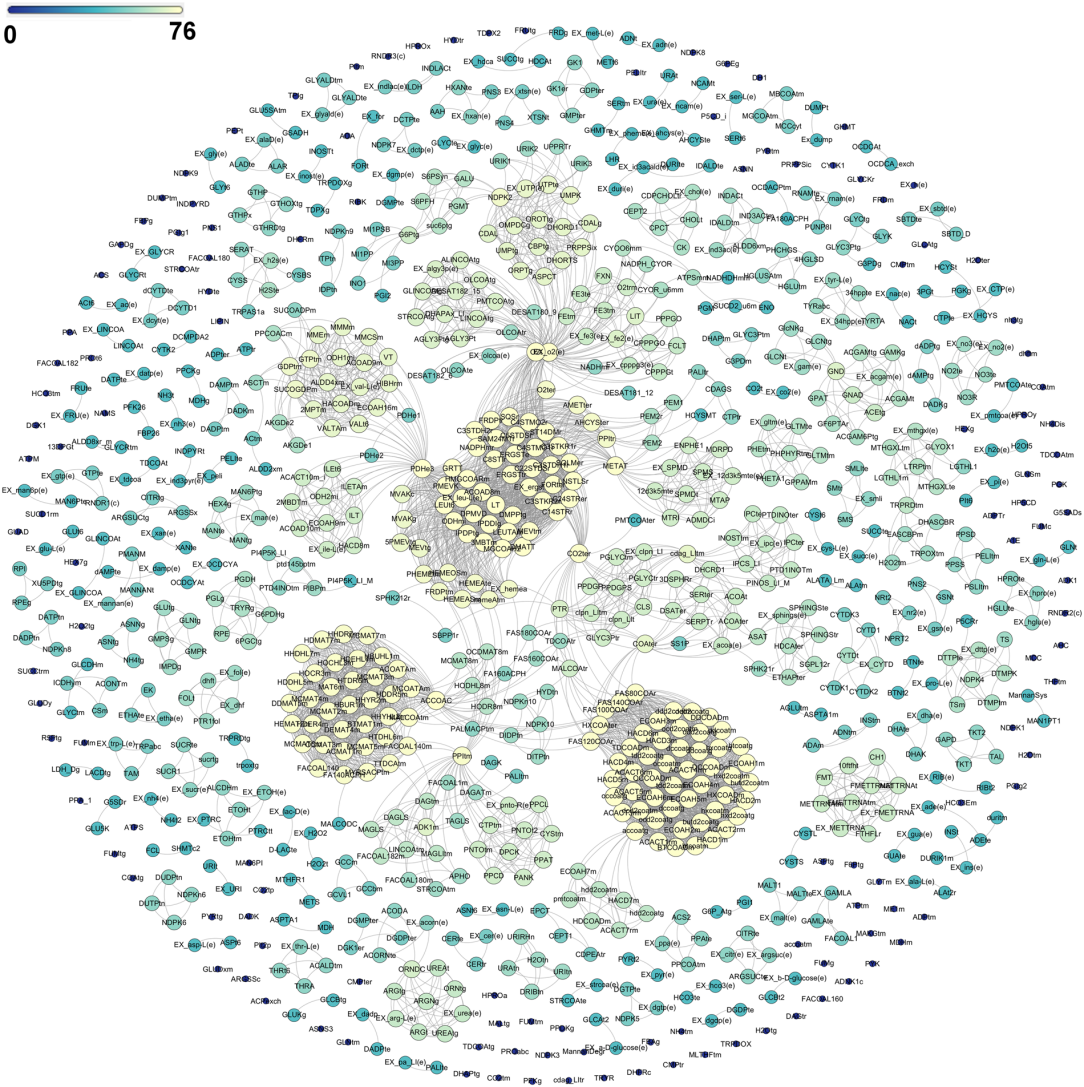
Network representation of the flux-coupled graph computed for the iAS556 metabolic network by flux coupling analysis. The network figure was created using the Fruchterman-Reingold algorithm implemented in Gephi version 0.8.2. Each node represents a reaction within the flux-coupled graph. Node sizes and colors were chosen according to the total number of reactions a particular reaction is coupled to, within the flux-coupled graph (degree of node within flux-coupled graph).

The above results demonstrate that structural constraints within the metabolic network confine the parasite to utilize specific environmental metabolites and direct it towards synthesis of various biomass metabolites. To answer the question, why the network might have been organized in a specific way, the flux-coupled subnetwork (Fig. 5, see Section 7 of Supplementary Information) derived from the whole genome-scale network was subjected to random perturbations while monitoring the re-organization of physiological flux coupling relationships within the network (see Methods). These flux relationships represent a complete set of coupled reactions within the *L. infantum* metabolic network, with respect to all the exchanges considered in the network. It was observed that the median number of fully and directionally coupled pairs reduced with the increase in number of simultaneous deletions (see Methods, Fig. S4A and B, Section 9 of Supplementary Information) and it requires at least 5 simultaneous deletions to notice a significant variation in their numbers. This suggests that reaction stoichiometry and reversibility impose constraints on the *L. infantum* metabolic network structure in such a way that only a few, specific reactions are tightly coupled to each other and rest of the reactions remain either isolated or coupled to fewer number of reactions. This lowers the chances of a coupled reaction set to be damaged by random errors. This is also supported by the fact that the flux-coupled graph generated from the unperturbed bipartite *L. infantum* iAS556 metabolic network contains few numbers of reactions [256] that have greater than average connectivity and a large number of reactions [693] with less than average connectivity (Average connectivity = 14.67; Supplementary Table S4). Also, the median number of reactions represented within modules remains unchanged with the increase in number of simultaneous deletions suggesting that the modular nature of the network is insensitive to random deletions (Fig. S4.C, Section 9 of Supplementary Information).

**Figure 6:**
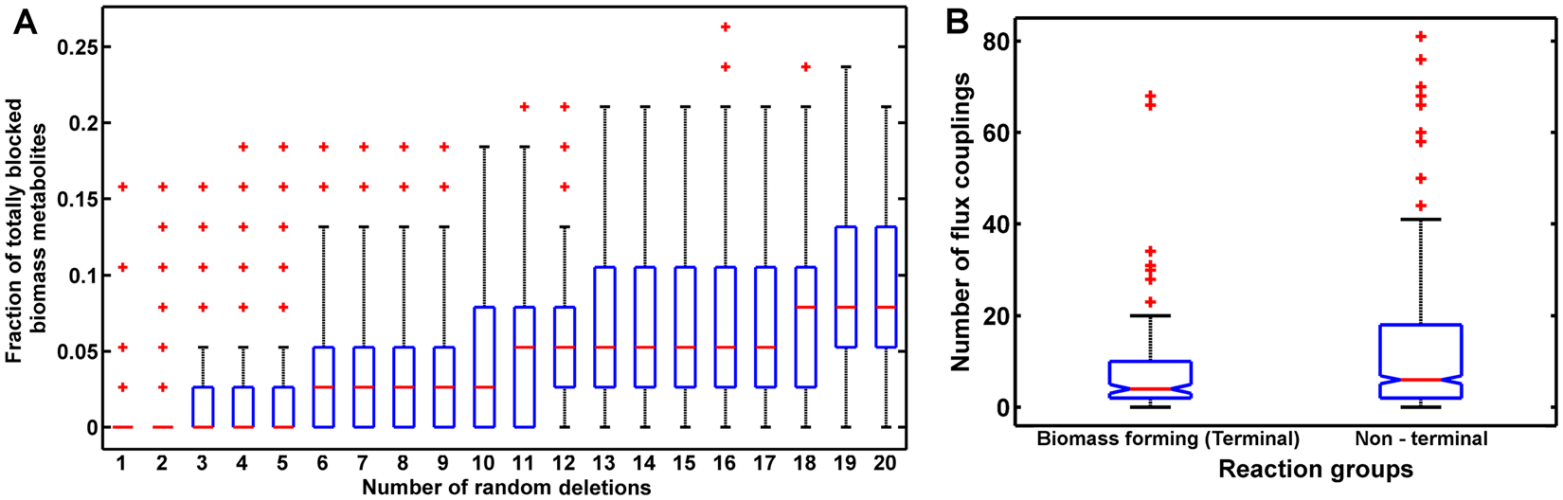
Robustness of physiologically coupled reactions within the *L. infantum* metabolic network to random deletions. (**A**) Boxplot indicating the distribution of the fraction of biomass metabolites that are totally blocked after each deletion or set of random deletions. (**B**) Notched boxplots for comparison of total number of flux couplings of biomass forming ultimate reactions (161 reactions) with all other non-terminal reactions (788 reactions) within the flux-coupled graph of the *L. infantum* JPCM5 metabolic network. The red colored points marked by ‘ + ’, indicate the outliers in each distribution.

We further wanted to investigate the biological significance of these specific couplings towards parasite metabolism and its effect on the biomass metabolites. To answer this, the fraction of biomass metabolites whose synthesis would be totally blocked after each sequential deletion, was calculated. The synthesis of a biomass metabolite was considered to be completely obstructed, if all the reactions forming that metabolite were deemed to be blocked after a sequential deletion (see Methods). The median percentage of metabolites expected to be blocked, also gradually increase with increasing number of reaction deletions; although only around 7% of biomass metabolites can be expected to be completely blocked after 20 sequential deletions (Fig. 6A). Also, the chance of obtaining more than 2% of metabolites to get completely blocked is higher (distribution skewed towards higher fractions) only if greater than 10 reactions are inhibited simultaneously (Fig. 6A). This suggests that a large number of less flux-coupled reactions observed within the network provide robustness to biomass-metabolite formation. Further, it seems that biomass-metabolite reactions are physiologically coupled to less number of reactions (median = 4) as compared to other non-terminal network reactions (median = 6), which on the other hand, tend to be coupled to a large number of other network reactions; although the fraction of variation explained by the difference between the biomass-forming vs. non-terminal reaction flux couplings is sufficiently small (Fig. 6B, W = 54107, Z = −2.9627, r = −0.09, *P = *0.003, Wilcoxon rank-sum test). This probably suggests that biomass-forming reactions are not blocked any more than other non-terminal reactions and that any effect is due to a handful of biomass reactions which demonstrate a low flux-coupling, rather than being a general effect specific to all biomass reactions. Also, 37 of the 40 biomass metabolites are formed by more than one reaction groups (connected components) within the flux-coupled graph, probably for unhindered production of the important biomass metabolites. The connected components and the corresponding pathways, in which each metabolite is involved, are given in Supplementary Table S5.

## Discussion

To summarize, a metabolic model for the *Leishmania infantum* JPCM5 parasite by considering extensive available information about metabolic reactions catalyzed by enzymes that span the entire genome is reconstructed newly. Flux analysis of parasite metabolism established that the parasite possesses a unique network of metabolic reactions whose functioning can be explained with respect to the stoichiometric and reversibility constraints imposed by the underlying metabolic network structure alone; the network structure being largely governed by the species-specific occurrence of enzymes in multiple subcellular locations (Section 2 of Supplementary Information). Comparison of predicted steady state reaction fluxes in *L. infantum* JPCM5 with^13^C isotope enrichment of metabolites in *L. mexicana* indicated that similar metabolic routes of glucose utilization are chosen within *L. mexicana* and *L. infantum* metabolism. Also, subtle differences in flux re-organization among the two species was observed, attributing to a probable indication of species-specific genes, multiple subcellular location of enzymes that provide new paths for flux redistribution, species-specific choice of external metabolites for satisfying metabolic demand and an increased drain of intermediate metabolites like non-essential amino acids, into the metabolic demand under a glucose-only situation. Thus, this result demonstrates the existence of a unique network structure within *Leishmania infantum* JPCM5 metabolism. Also, the metabolic states within the promastigote and amastigote scenarios were predicted unde﻿r appropriate uptake constraints. Mainly, it was observed that fatty acids, amino sugars and mannose can compensate for reduced glucose uptake within the amastigote. Fatty acids produce intracellular glutamate, whereas amino sugars and mannose satisfies the production of intracellular aspartate and mannan, respectively.

The organization of *L. infantum* metabolism, though simple, can be dynamic enough to re-route external metabolites towards synthesis of specific internal biomass metabolites. This dynamic nature is due to the non-essential amino acid motif-based reaction sets within the metabolic network that get coupled with respect to various combinations of environmental metabolites. The dynamic non-essential amino acid motif diverts specific input metabolites towards specific outputs and constrains the utilization of diverse resources simultaneously. This analysis also suggests that glucose and tyrosine alone cannot produce other biomass metabolites, and their role is to only supplement the quantities of output metabolites formed through non-essential amino acids, indicating a tight coupling between glucose, tyrosine and non-essential amino acids. This constrained distribution is largely influenced by the presence of subcellular compartments that restrict coupling of specific reactions to achieve redox or ATP balance. Glycosome restricts the transfer of ADP between the glycosomal reactions and the cytoplasmic reactions, thereby inducing a redox coupling between trypanothione reductase and pentose phosphate shunt, and upper and lower part of glycolysis in the promastigote. Similarly, due to reduced glucose uptake observed in the amastigote, NAD redox coupling between fatty acid *β*-oxidation and lower part of glycolysis is established. The role of mitochondrion on the other hand, is to redirect C4 dicarboxylic acids produced from glycolysis and non-essential amino acid motif towards TCA cycle for formation of mitochondrial glutamate, which regenerates reducing equivalents for oxidative phosphorylation.

The *L. infantum* flux-coupled network structure is highly modular and assortative, thereby remaining robust to a random node failure^30, 31^. This also suggests that the modular nature of the network and presence of enzymes in dual/multiple subcellular locations are important characteristics of *L. infantum* network that provide adaptation to the changing environment. The network robustness ensures an unobstructed production of biomass metabolites. Furthermore, the benefit of the network structure being robust to random mutations is not only gained by biomass-forming reactions but also equally by all the other network reactions. Few biomass metabolites like ergosterol, heme A, isoleucine, leucine, valine, pantothenate, and zymosterol, are produced only from a set of specific enzymes, which form a highly connected, single connected component (central within the flux coupled subnetwork, Fig. 5), which might be useful as drug targets. Enzymes of heme and sterol metabolism in *Leishmania* have already been indicated as important targets^32, 33^. All our results thus, indicate a rigid yet flexible metabolic milieu of *Leishmania* metabolism that is well adapted to its microenvironment, and demonstrate the probable metabolic strategies utilized by the parasite for its survival. Further, the aforementioned analyses of the proposed model also provide a catalogue of experimentally testable hypotheses implicated for the first time in *L. infantum* metabolism. These model-based analyses can thus, pave ways to design suitable experiments that can explore the different possibilities of metabolic adaptation of *L. infantum* within the host. These leads may further help the human community to combat against this neglected, but deadly tropical parasitic infection.

## Methods

### Model reconstruction

The up-to-date iAS556 genome-scale metabolic network of *L. infantum* was reconstructed using our established strategy standardized for reconstruction of the *L. infantum* energy metabolic network^15^. Genes for the iAS556 network reconstruction were obtained for *Leishmania infantum* JPCM5 genome (assembly ID: GCA_000002875.2 ASM287v2), updated as of 16/12/2011. See Sections 1 and 2 of Supplementary Information for details.

### Model analysis

#### Flux balance analysis

Representing the reconstructed network as a constraint-based model with appropriate intracellular and exchange constraints, flux balance analysis (FBA)^34^ was performed on the iAS556 network to predict metabolic fates of given input metabolite exchanges, while optimizing for a *Leishmania*-specific metabolic demand reaction. Details of the constraints applied on the exchange & intracellular reactions are given in Section 4 of Supplementary Information.

#### Flux coupling analysis

To identify physiological flux coupling relationships (flux-coupled subnetwork) within the iAS556 network, flux coupling analysis (FCA)^24, 35^ was performed, while considering the presence of all input exchanges. Flux coupling analysis is a constraint-based procedure that utilizes the stoichiometric and reversibility information of reactions within the metabolic network to identify flux dependencies between a pair of reactions, given specific environmental (exchange) constraints. If a non-zero flux in one reaction implies a non-zero flux in the other reaction then the two reactions are coupled with each other. To obtain an actual sense of the coupled fluxes and to avoid artificial coupling of large set of reaction fluxes with the metabolic demand, the metabolic demand reaction was removed from the network while including independent reversible drains for the demand metabolites in the network. See Section 8 of Supplementary Information for details.

### Model validation with experimental data

#### Utilization of carbon substrates

The fate of carbohydrates, like glucose (Glc) and mannose (Man), amino sugars like N-acetylglucosamine (Acgam), non-essential amino acids, like alanine (Ala), aspartate (Asp), glutamate (Glu) and hexadecanoate fatty acids (Hdca), was predicted for both the stages by computing the steady state fluxes for each reaction under uptake of each carbon source one at a time, while optimizing for the metabolic demand reaction. Exchanges of proline (Pro), glycine (Gly), essential amino acids, like serine (Ser), threonine (Thr), leucine (Leu), isoleucine (Ile), valine (Val), lysine (Lys), phenylalanine (Phe), were all provided simultaneously for all the other amino acids (AA) situation. The fate of the mentioned metabolites was specifically studied in order to compare the model predictions with observations from experimental ^13^C-isotope labeling profiles of the above metabolites generated in different developmental stages of *L. mexicana* ^5^. The ^13^C isotope labeling profiles in this experimental study were obtained by growing *L. mexicana* promastigotes and amastigotes within a chemically defined growth medium (CDM) containing specific ^13^C -tagged carbon sources and analyzing the isotope enrichment of ^13^C labeled intracellular metabolite pools using gas chromatography-mass spectrometry (GC-MS).^13^C isotope enrichment measures the amount of incorporation of ^13^C tagged environmental metabolite (in mol percent) into its intracellular catabolic products, thereby indicating the preference of utilization of a particular metabolite through specific pathways.

The *in-silico* stoichiometric-based utilization of each metabolite produced from previous reaction into subsequent reaction/s within the iAS556 network was traced by the contribution of previous reaction flux into its subsequent reaction flux. Statistical comparison of model predictions with experimental data was performed for the glucose-only situation as – i) only glucose is catabolized by a large number of steps (sufficient sample size for comparison) each of which is labeled from ^13^C isotope-resolved metabolomics and, ii) because the normalized ^13^C isotope enrichment values were reported for only labeled glucose. Spearman rank correlation was computed to identify the strength of associativity between predicted reaction steady state fluxes (normalized to flux of hexokinase) and ^13^C isotope enrichment values of metabolites (normalized to glucose-6-phosphate) grown on labeled ^13^C glucose^5^. In all the mentioned conditions, uptake of glucose, essential amino acids, cofactors and ions were always kept unconstrained irrespective of change in environment, as they are absolutely essential for growth. Also, drains for overflow metabolites were provided in the model to release by-products.

#### Reaction knockout analyses

Single reaction knockout phenotypes that correspond to a zero flux through the metabolic demand were predicted by constraining the upper and lower bounds of each target reaction to zero flux and performing subsequent optimization. The model-predicted knockout phenotypes were predicted with experimentally determined *Leishmania*-specific knockout phenotypes. See Section 6 of Supplementary Information for details.

### Sensitivity analysis

To understand the role of non-essential amino acids, a sensitivity analysis was performed to understand the effect of glucose and uptakes of all the amino acids, while optimizing for the formation of glutamate, alanine, aspartate, glutamine, proline, glycine, myoinositol and mannogen, each formation considered as a separate objective function. The uptake of glucose and choice of myoinositol and mannogen synthesis as objectives is to understand and compare the coherent role of glucose with amino acids. Due to the role as intermediate by-products of reactions that are consumed for optimizing metabolic demand, for each simulation, drain (secretion/release) for alanine, glutamate, glutamine and proline was considered. Each simulation was performed separately, considering one input at a time while constraining all other exchanges to zero. All remaining exchanges in the model were kept default (lower bound = −1000 and upper bound = 1000)^36^.

The considered input uptakes can be given by x, where x $\in$ A = {Asn, Ala, Arg, Glc, Cys, Gln, Glu, His, Ile, Asp, Leu, Lys, Met, Phe, Pro, Ser, Thr, Trp, Tyr, Val}. Four different scenarios were considered for optimizing the considered objective functions namely,

i) only one input source (x) constrained to a non-zero flux value (lower bound = −1000) at a time and all other inputs (A\{x}) are constrained to zero;

ii) two inputs given at a time – glucose which is kept fixed and a variable second input (x) with non-zero flux given one at a time and all other inputs are constrained to zero;

iii) two inputs given at a time – one being isoleucine which is kept fixed and a variable second input (x) constrained to a non-zero flux value given one at a time and all other inputs are constrained to zero;

iv) three inputs given simultaneously – glucose, tyrosine which are fixed and a variable third input (x) with non-zero flux given one at a time and all other inputs are constrained to zero.

### Effect of subcellular compartmentalization on flux distribution

For exploring the role of subcellular compartments in reinforcing coupling relationships, reactions occurring in specific subcellular compartments like glycosome or mitochondrion, were shifted to the cytoplasm, while deleting the corresponding compartment from the iAS556 model. Also, it was ensured that, reactions occurring in dual or multiple locations (for example, glycosome and cytoplasm or mitochondrion and cytoplasm) were considered as a single reaction in cytoplasm to avoid redundancy in the re-created model.

### Reconstitution of flux relationships under random perturbations

Scenarios for perturbation of flux-coupled relationships due to random reaction malfunction were created by deleting random single or multiple reactions while re-performing FCA on the network for each case. 1000 random simulations were performed for each case. Each deletion represents the complete loss of function of a given gene or sets of genes absolutely eliminating the role of their corresponding reactions.

## Electronic supplementary material

Supplementary Information

Supplementary Data S1

Supplementary Data S2

Supplementary Table S1

Supplementary Table S2

Supplementary Table S3

Supplementary Table S4

Supplementary Table S5
