# Supplementary material for: Revealing the mystery of metabolic adaptations using a genome scale model of Leishmania infantum
Source: Sci Rep. 2017 Aug 31;7:10262. doi: 10.1038/s41598-017-10743-x (PMC5579285; doi:10.1038/s41598-017-10743-x)
Supplement: Supplementary file 1 — Supplementary Information [file 41598_2017_10743_MOESM1_ESM.pdf]

SUPPLEMENTARY INFORMATION

for

Revealing the mystery of metabolic adaptations using a genome scale model of *Leishmania infantum*

Abhishek Subramanian[1,2], Ram Rup Sarkar[1,2]*

[1] Chemical Engineering and Process Development, CSIR-National Chemical Laboratory, Pune, Maharashtra, India

[2] Academy of Scientific & Innovative Research (AcSIR), CSIR-NCL Campus, Pune, India

*Corresponding Author:

Ram Rup Sarkar,

Chemical Engineering and Process Development, CSIR-National Chemical Laboratory, Pune, Maharashtra, India

Tel: +91-20-2590 3040; Fax: +91-20-2590 2621; E-Mail: rr.sarkar@ncl.res.in

Contents

1. **Model reconstruction……………………………………………………..2**
2. **Model properties……………………………………………………………3**
3. **The metabolic demand reaction…………………………………….....9**
4. **Flux balance analysis..………………………………………………….…..11**
5. **Fate of carbon sources within network…...………………….………13**
6. **Reaction knockout analysis…………………………………………..15**
7. **Role of subcellular compartmentalization……………………….....19**
8. **Flux coupling analysis (FCA)………………………………………....22**
9. **Perturbation of physiological coupling relationships……...........24**
10. **Additional Files (Legends)....…………………..............................25**

1. Model reconstruction

A previously established strategy standardized for reconstruction of *L. infantum* energy metabolic network[1], was used for iAS556 model reconstruction. Further, the iAS142 *L. infantum* energy metabolic network[1] was expanded to include the entire genome-scale metabolism.

a) Identification and assignment of reactions to subcellular locations: Genes for the iAS556 network reconstruction were obtained for *Leishmania infantum* JPCM5 genome (assembly ID: GCA_000002875.2 ASM287v2) updated as of 16/12/2011. Based on previously reported facts regarding *Leishmania* metabolism and *in-silico* prediction of signals within the gene sequences, reactions were associated with its genes and assigned to their requisite subcellular locations (Supplementary Data S1 for the information curated). For each reaction in the model a confidence score was assigned. The concept of the confidence score has been previously introduced[1]. A high confidence score suggests that the reaction is strongly supported by both experimental and *in-silico* evidence for its occurrence.
b) Incorporation of transports and exchanges: The transports and exchange reactions of previously reported carbon and nitrogen sources namely, carbohydrates, amino acids, fatty acids, amino sugars, purines and pyrimidines; fermentation products like pyruvate, lactate, acetate and succinate; vitamins folate, pantothenate, nicotinate, pyridoxine, protoheme and biopterin, other vital sources like iron, nitrates, nitrites, CO_2, H_2O, H_2S, HCO_3 and NH_3; and ions/protons were also included. Missing intracellular reactions and transports were added through the iterative refinement procedure.
c) Model iterative refinement: The primary metabolic network thus formed, was iteratively refined to identify missing gaps by comparing with observations in *Leishmania* literature. Applying constraints specific to *Leishmania* metabolism, the primary model was tested for predictions of known knockout phenotypes, prediction of overflow metabolites reported in *Leishmania* literature, matching predicted profiles with the experimental ^{13}C targeted metabolomics studies reported for related *L. infantum* or *Leishmania* species[2], comparison with previously published *Leishmania* metabolic networks and qualitative mechanistic observations discretely reported in *Leishmania* literature. If there were discrepancies in predictions, the gaps were identified [either novel enzymes (maybe intracellular metabolic reaction or transport) or novel subcellular locations of existing enzymes] and filled. The

modified network was again refined through the above procedure until the network is sufficiently able to reproduce the observations reported for *Leishmania* metabolism. Accordingly, reactions in appropriate subcellular reactions (for example, novel placement of fatty acid oxidation enzymes in glycosome for NAD redox coupling with glycolysis) and novel reactions (like threonine aldolase [UniProt ID: A4HRH1], which was previously annotated as an uncharacterized protein within the *L. infantum* JPCM5 genome) were identified.

2. Model properties

Known biological information and *in-silico* prediction of reaction subcellular locations and functions were integrated collectively to obtain a gene-protein-reaction (GPR) framework specific to the metabolism of *L. infantum*. The GPR framework was constructed with respect to reactions involved in production and transport of metabolites by following a stringent reconstruction strategy considering heterogeneous data, tools, supporting analysis and the assigned cellular compartments[1]. A list of reactions, their corresponding genes, enzymes, and metabolites were compiled with appropriate literature support, in combination with sequence analysis to substantiate evidence related to reactions, its biological significance, and location. These metabolites and the corresponding reactions were distributed into 9 different model compartments i.e. glycosome, cytosol, mitochondrion, mitochondrial inter-membrane space endoplasmic reticulum, acidocalcisome, vacuole, nucleus and extracellular space. All information was organized in the rBioNet toolbox[3] to eventually obtain an extensive model reconstruction, iAS556 for *L. infantum* genome-scale metabolism. The model is made available in the SBML format (Supplementary Data S2), for reference. Using the established convention of naming *in silico* constraint based models; the present computational model was named 'iAS556'. The model name commenced with 'i' indicating *in silico*, followed by the first author's first and last initials ('AS') and followed by the number of genes, i.e. 556 genes that are part of the model.

Accordingly, the iAS556 metabolic network consists of 1260 total reactions spanning the 9 different subcellular compartments. Out of the 1260, around 645 metabolic reactions, 478 transport, 136 exchange reactions, and a metabolic demand reaction constitute the iAS556 network. A total of 647 reactions could be associated with 556 genes from the *L. infantum*

genome (Table A). More importantly, the metabolic demand reaction considered in generating the present GPR network varied from the biomass drain reactions employed by previous similar studies since it was formulated using relative availability of metabolites in the log phase promastigotes as detected from metabolomics experiments[2] [See Section 4 for details]. There are 136 exchange reactions included in the model, which is a high number compared to the previous genome-scale reconstructions in Trypanosomatids. This can be attributed to the presence of exchanges of comprehensive set of metabolites that are experimentally known to be salvaged by the parasite from the host environment.

Carbohydrate metabolism constitutes approximately 7% of total reactions in the model. Amino acid metabolic pathways represented a major portion of around 12% of the total, demonstrating their importance in metabolism of *Leishmania* (Figure S1.A). The iAS556 metabolic network encompasses the full genome of *L. infantum* majorly comprising of 25% cytosolic, 15% mitochondrial and 9% of glycosomal & endoplasmic reticulum reactions (Figure S1.B).

Table A: Properties of the *L. infantum* iAS556 network reconstruction

Property	Count
Genes	556
Reactions	1260
(i) Gene associated (intracellular)	623
(ii) Gene associated (transport)	24
(iii) Non-Gene associated (intracellular)	22
(iv) Non-Gene associated (transport)	454
(v) Exchange	136
(vi) Demand (Metabolic)	1
Metabolites	1160
Compartments	9
Literature References (for reconstruction) [See Supplementary Data S1]	~160
Databases Consulted (for reconstruction) [1]	10

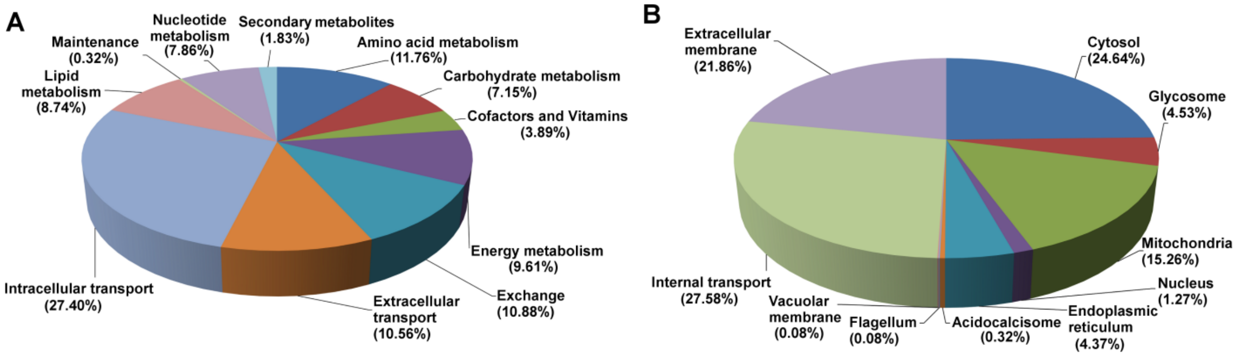

Figure S1 - Properties of the iAS556 model - A) Classification of model reactions into their metabolic pathways, B) Classification of model reactions into their compartments.

Additionally, around 49% of the total reactions were present in membranous compartments representing the intra-cellular and extracellular transport reactions for various metabolites.

The iAS556 reconstruction was compared with the *L. major* iAC560 constraint-based model[4]. Around 29% of the model reactions (excluding exchange reactions) are unique to the iAS556 model and have been newly curated. The comparisons indicate that the iAS556 model accounts for 223 novel intracellular reactions (35 compartmental + 188 intracellular transports). These differences arise due to inappropriate assignment of reaction subcellular locations, unavailability of appropriate information for certain reactions, absence/existence of multiple subcellular location of enzymes, absence of important intracellular transport reactions reported through literature, and absence of mitochondrial inter-membrane space compartment within the iAC560 model, when compared to iAS556 model. These major gaps have been filled in the iAS556 metabolic network. Also, one of the major updates within the *L. infantum* iAS556 model was the occurrence of few fatty acid enzymes (that convert NAD to NADH) within the glycosome, which was previously presumed in other reconstructions to occur within the cytoplasm. The reason for this placement was the NAD redox coupling that exists between fatty acid oxidation and glycolysis. This was also supported through proteomics experiments in *L. donovani*[5]. The differences in intracellular reaction subcellular location between the two models are enlisted in Table B. The confidence score for these reactions have also been indicated to emphasize the confidence with which their subcellular locations were determined[1]. A confidence score of 5 indicates the strongest evidence for a reaction to exist within the model, whereas a score of 1 indicates the lowest confidence.

Table B: Comparison of reaction subcellular locations between iAS556 with the iAC560 model

Reaction	Abbreviation	iAC560	iAS556	Confidence Score in iAS556
4-Coumarate:CoA ligase (AMP-forming)	4COUCOAL, 4COUCOALm	cytosol	cytosol	2
		--	mitochondria	2
L-4-hydroxyglutamate semialdehyde dehydrogenase	4HGLSD	mitochondria	cytosol	2
aldose 1-epimerase	A1E	cytosol	cytosol	2
		glycosome	--	
acetyl-CoA carboxylase	ACCOAC	mitochondria	cytosol	2
aconitate hydratase	ACONTc, ACONTm	--	cytosol	4
		mitochondria	mitochondria	5
acetyl-CoA synthetase	ACS, ACS2	mitochondria	cytosol	5
		mitochondria	cytosol	2
adenosine kinase	ADNK1c	cytosol	cytosol	5
		endoplasmic reticulum	--	
		mitochondria	--	
		glycosome	--	
1-acylglycerol-3-phosphate O-acyltransferase	AGPATr	mitochondria	endoplasmic reticulum	2
acetyl-CoA:1-alkyl-sn-glycero-3-phosphate 2-O-acetyltransferase	AKG3PAT_LI	cytosol	glycosome	2
aldehyde reductase	ALDR	cytosol	mitochondria	2
argininosuccinate synthase	ARGSSc, ARGSSx	cytosol	cytosol	2
		--	glycosome	
alanine aminotransferase	ALATA_Lm	cytosol	--	5
		mitochondria	mitochondria	
carbon-dioxide:ammonia ligase (ADP-forming,carbamate-phosphorylating)	CDAL, CDALg	cytosol	cytosol	5
		--	glycosome	5
choline/ethanolamine phosphotransferase	CEPT1, CEPT2	cytosol	endoplasmic reticulum	1
		cytosol	endoplasmic reticulum	1
citrate Synthase/lyase	CSc, CSm	--	cytosol	5
		mitochondria	mitochondria	5
cytidylate kinase (dCMP)	CYTK2n	nucleus	cytosol	1
dephospho-CoA kinase	DPCK	cytosol	mitochondria	2
diphosphomevalonate decarboxylase	DPMVD	glycosome	--	5
		cytosol	cytosol	
deoxyuridine triphosphatase	DUTPDPn	mitochondria	--	2
		nucleus	nucleus	
cis-2-Methyl-5-isopropylhexa-	ECOAH13m	glycosome	--	2

2,5-dienoyl-CoA hydro-lyase		mitochondria	mitochondria	
trans-2-Methyl-5-isopropylhexa-2,5-dienoyl-CoA hydro-lyase	ECOAH14m	glycosome	--	2
		mitochondria	mitochondria	
ferulate:CoA ligase (AMP-forming)	FERCOAL, FERCOALm	cytosol	cytosol	2
		--	mitochondria	2
fumarase	FUMc, FUMg, FUMm	--	cytosol	5
		glycosome	glycosome	3
		mitochondria	mitochondria	4
fatty-acid--CoA ligase	FACOAL140m, FACOAL160m, FACOAL180m, FACOAL182m, FACOAL1m, FACOAL2m	cytosol	mitochondria	2
L-glutamate 5-semialdehyde dehydratase (spontaneous)	G5SADs	cytosol	cytosol	2
		mitochondria	--	
glucose 6-phosphate dehydrogenase	G6PDHg	cytosol	--	5
		glycosome	glycosome	
glycine cleavage complex	GCCbm	cytosol	--	4
		mitochondria	mitochondria	
glycine hydroxymethyltransferase	GHMT, GHMTm	cytosol	cytosol	5
		--	mitochondria	5
alcohol dehydrogenase	GLCDHm	cytosol	mitochondria	1
glutamine synthetase	GLNSm	cytosol	mitochondria	2
GMP synthase (glutamine-hydrolysing)	GMPS2, GMPSg	cytosol	cytosol	4
		--	glycosome	4
hydroxymethylglutaryl-CoA reductase	HMGCOArm	glycosome	--	5
		mitochondria	mitochondria	
N-acetylglucosamine-6-phosphate deacetylase	GNAD	cytosol	glycosome	5
Guanine phosphoribosyltransferase	GPRTg	cytosol	glycosome	5
L-glutamate 5-semialdehyde dehydrogenase	GSADH	mitochondria	cytosol	2
ubiquinone biosynthesis monooxygenase Coq7	H3MS2	mitochondria	cytosol	2
4-Hydroxy-2-ketopimelate aldolase	HKA	mitochondria	cytosol	2
3-hydroxy-1-pyrroline-5-carboxylate dehydrogenase	HP5CD	mitochondria	cytosol	2
L-hydroxyproline reductase (NAD)	HPROa	cytosol	cytosol	2
		mitochondria	--	
L-hydroxyproline dehydrogenase (NAD)	HPROx, HPROy	mitochondria	cytosol	2
		mitochondria	cytosol	2
isocitrate dehydrogenase (NADP+)	ICDHyc, ICDHym	--	cytosol	5
		mitochondria	mitochondria	5

isopentenyl-diphosphate D-isomerase	IPDDIg	cytosol	--	2
		glycosome	glycosome	
di-trans,poly-cis-Decaprenyl-diphosphate:isopentenyl-diphosphate undecaprenylcistransferase	IPDPUPT	cytosol	mitochondria	2
inositol-1,3,4,5-trisphosphate 5-phosphatase	IPP5P2m, IPP5Pm	cytosol	mitochondria	2
		cytosol	mitochondria	2
D-lactate dehydrogenase	LDH_Dg	cytosol	--	
		glycosome	glycosome	2
methylcrotonoyl-CoA carboxylase	MCC, MCCcyt	mitochondria	mitochondria	2
		--	cytosol	2
malic enzyme (NADP)	ME1m	cytosol	mitochondria	2
methylglutaconyl-CoA hydratase	MGCOAH2	cytosol	mitochondria	2
pyrroline-5-carboxylate reductase	P5CRr	cytosol	cytosol	2
		mitochondria	--	
methylene-fatty-acyl-phospholipid synthase	PEM1	cytosol	endoplasmic reticulum	3
phosphatidyl-N-methylethanolamine N-methyltransferase	PEM2, PEM2r	cytosol	endoplasmic reticulum	1
		cytosol	endoplasmic reticulum	1
glucose-6-phosphate isomerase	PGI1, PGI2, PGIg1,PGIg2	cytosol	cytosol	4
		cytosol	cytosol	4
		--	glycosome	5
		--	glycosome	5
L-1-Pyrroline-3-hydroxy-5-carboxylate (spontaneous conversion to L-4-hydroxyglutamate semialdehyde)	PHCHGS	cytosol	cytosol	2
		mitochondria	--	
phenylalanine transaminase	PHETA1	cytosol	cytosol	5
		mitochondria	--	
1-phosphatidylinositol-4,5-bisphosphate phosphodiesterase	PI45BPP_LI, PI45BPP_LI_M	cytosol	cytosol	2
		--	mitochondria	2
phosphatidylinositol 4-phosphate 5-kinase	PI4P5K_LI, PI4P5K_LI_M	cytosol	cytosol	2
		--	mitochondria	2
1-phosphatidylinositol 3-kinase	PIN3K_LI, PIN3K_LI_M	cytosol	cytosol	1
		--	mitochondria	2
phosphatidylinositol synthase	PINOS_LI_M	cytosol	mitochondria	1
phosphomevalonate kinase	PMEVK	cytosol	cytosol	5
		glycosome	--	
phosphopantothenoylcysteine decarboxylase(hypothetical protein)	PPCD	cytosol	mitochondria	1
phosphopantothenate--cysteine ligase	PPCL	cytosol	mitochondria	2

propionyl-CoA carboxylase	PPCOAC, PPCOACm	--	cytosol	2
		mitochondria	mitochondria	2
phosphatidylserine decarboxylase	PPSD	cytosol	mitochondria	5
phosphatidylserine synthase	PPSS	cytosol	cytosol	
		mitochondria	mitochondria	2
phosphomannomutase	PMANM	cytosol	cytosol	5
		glycosome	--	
ribonucleoside-diphosphate reductase	RNDR1(c), RNDR1(n), RNDR2(c), RNDR2(n), RNDR3(c), RNDR3(n), RNDR4(c), RNDR4(n)	--	cytosol	5
		nucleus	nucleus	5
		--	cytosol	5
		nucleus	nucleus	5
		--	cytosol	5
		nucleus	nucleus	5
		--	cytosol	5
		nucleus	nucleus	5
sinapate:CoA ligase (AMP-forming)	SINCOAL, SINCOALm	--	cytosol	2
		mitochondria	mitochondria	2
squalene synthase	SQSm, SQSr	glycosome	mitochondria	4
		cytosol	endoplasmic reticulum	4
3,4-Dihydroxy-trans-cinnamate:CoA ligase (AMP-forming)	TCAFCOAL, TCAFCOALm	cytosol	cytosol	2
		--	mitochondria	2
trans-Cinnamate:CoA ligase (AMP-forming)	TCINCOAL, TCINCOALm	cytosol	cytosol	2
		--	mitochondria	2
tetrahydrofolate:L-glutamate gamma-ligase (ADP-forming)	THFGLUS	cytosol	mitochondria	2
trypanothione reductase	TRYR, TRYRg, TRYRn,	--	cytosol	5
		glycosome	glycosome	5
		--	nucleus	5
undecaprenyl-diphosphate synthase	UDPDPS	cytosol	mitochondria	2
uridine nucleosidase	URIRHn	nucleus	cytosol	5

3. The metabolic demand reaction

To perform flux balance analysis (FBA) of this genome-scale metabolic network, a *Leishmania*-specific metabolic demand reaction was formulated using relative metabolite signal intensity data gathered from a published untargeted metabolomics experiment carried out in *L. donovani*, a visceral *Leishmania* species evolutionarily related to *L. infantum*[2]. The metabolites considered for formulating the demand reaction were those that were included in the *L. major* iAC560

biomass objective function[4], except for putrescine, spermidine, sphingomyelin, cardiolipin and phosphatidylinositol. Putrescine and spermidine are required to produce reduced trypanothione, which is a part of the demand reaction and hence, were not separately included. Sphingomyelin and cardiolipin were not detected in the above experiment and hence, not included in the demand reaction. Sphingolipids are not essential for growth in *Leishmania* and most of them are acquired after integration with host membranes[6]. Cardiolipin is found in very low concentrations in *Leishmania* and its functions in *Leishmania* are not very well understood[6]. Phosphatidylinositol is required to form diacylglycerol, which is included in the demand reaction and hence, was not included separately. Also, phosphatidylinositol is present in low detectable concentrations within the cell membranes of *Leishmania*[6]. It is also still debated whether phosphatidylserine is acquired from the host or *Leishmania* synthesizes it.

Previous constraint-based models in Trypanosomatids have not used organism specific metabolomics data for this purpose[4,7]. Maximizing the flux of this hypothetical reaction was considered as the objective for performing flux balance analysis (FBA).

Table D: Metabolites chosen for metabolic demand and coefficients

**Metabolite in demand reaction**	**Metabolite abbreviation in the model**	**Identified metabolite in experiment[2]**	**Available metabolite fraction in total metabolite pool (Coefficient of metabolite in demand reaction)**
serine	ser_L	serine	0.01056
arginine	arg_L	arginine	0.00454
cysteine	cys_L	cysteine	0.00034
glutamic acid	glu_L	glutamic acid	0.03294
glutamine	gln_L	glutamine	0.01606
glycine	Gly	glycine	0.0001
histidine	his_L	histidine	0.00384
isoleucine	ile_L	isoleucine/leucine	0.05987
leucine	leu_L	isoleucine/leucine	0.05987
aspartic acid	asp_L	aspartic acid	0.00745
lysine	lys_L	lysine	0.00486
methionine	met_L	methionine	0.00882
phenylalanine	phe_L	phenylalanine	0.02738
proline	pro_L	proline	0.05032
threonine	thr_L	threonine	0.00642
asparagine	asn_L	asparagine	0.01606
trypanothione	Trprd	trypanothione	0.00036

alanine	ala_L	alanine	0.01467
tryptophan	trp_L	tryptophan	0.00092
tyrosine	tyr_L	tyrosine	0.00872
valine	val_L	valine	0.03742
AMP	AMP	AMP	0.0011
CMP	CMP	CMP	0.00004
GMP	GMP	GMP	0.00006
UMP	UMP	pseudouridine-5-phosphate, UMP	0.0001
dCMP	dCMP	assumed same as CMP	0.00004
dGMP	dGMP	assumed same as dCMP	0.00004
dTMP	dTMP	thymine	0.00022
dAMP	dAMP	assumed same as dTMP	0.00022
diacylglycerol	dag_LI	DAG(36:4)	0.0003
triacylglycerol	tag_LI	TAG(54:7)	0.00008
ergosterol	Ergst	ergosterol	0.00031
phosphatidylcholine	pc_LI	lysophosphatidylcholine(20:3/1)	0.00086
phosphatidylethanolamine	pe_LI	lysophosphatidylethanolamine(18:2/1)	0.0001
zymosterol	Zymst	zymosterol, cholestadienol, 7-dehydrocholesterol	0.00006
heme	hemeA	porphobilinogen	0.00065
pantothenol	pnto_R	pantothenol	0.00018
mannan	Mannan	glycogen, stachyose, maltotetraose	0.0002

4. Flux balance analysis

To predict the distribution of metabolite fluxes through the network, the iAS556 network was represented in the form of a stoichiometric matrix consisting of 1260 rows (reactions) and 1160 columns (metabolites). Applying stoichiometric and reversibility constraints, the network was represented as a constraint - based model and steady state fluxes through model reactions were predicted using FBA[8]. Flux balance analysis was performed using COBRA toolbox version 2.0[9] as implemented in MATLAB R2012a and the 'glpk' solver. Irreversible reactions were constrained to occur in a specific direction from reactant to product. Uptake reactions were constrained irreversibly such that metabolites are consumed from the environment whereas secretions of metabolites are constrained such that metabolites are released into the environment. Exchanges for few metabolites that can be both utilized and produced (for example, alanine) were constrained reversibly. Uptake reactions in promastigote stage of the parasite were constrained such that all the uptakes of different carbon and nitrogen sources were sufficiently available whereas, the same uptake reactions in amastigotes were constrained to sub-optimal

rates because uptake of glucose and amino acids is known to be reduced in this stage[10]. Also, in both stages, proline is typically formed from glutamate rather than uptake from environment. The amastigotes are also characterized by hypoxic environmental conditions with reduced glucose and amino acid uptake; and a unique fatty acid uptake chosen as an alternative to compensate for unavailability of glucose[10,12]. These constraints on the uptakes were applied so that fatty acids would be optimally selected to compensate for low glucose uptake in amastigotes and overflow metabolite secretion would be relatively high in promastigotes as compared to amastigotes[10]. As flux changes in the metabolic network are governed only by constraints on uptake of environmental metabolites, only the bounds on exchange fluxes were varied while fixing bounds of important internal reactions and transports to make them irreversible, according to the information obtained from *Leishmania* literature[10,12]. Majority of the internal reactions were fixed at default bounds (lower bound = -1000 and upper bound = 1000)[11]. The exchange flux constraints applied to generate the two metabolic states (promastigote and amastigote) and simulated uptake rates of different metabolites in both these stages are given in Table C. Information related to all the bounds are given in Supplementary Data S2.

Stoichiometrically balanced cycles were systematically identified by tracing the flux distributions of the reactions from the input exchanges. To eliminate these cycles, few important transports, like ATP & ADP within mitochondria, transport of overflow metabolites like lactate, etc. were kept irreversible to direct the metabolites into/away from a subcellular compartment/cell as per the knowledge of specific input and output/overflow metabolites reported in *Leishmania* literature. For example, glucose is consumed from the environment into glycolysis to form succinate, which is utilized within the TCA cycle. Also, in some cases like the pentose phosphate pathway for example, although the bounds of the intra-compartmental transports was kept reversible, once the metabolites enter into a compartment, it was ensured that they are irreversibly catabolized and converted into another metabolite within the compartment. The irreversibility of internal reactions was also fixed according to the knowledge of either the metabolite flux distribution or production of specific output metabolites as reported in *Leishmania* literature. All the simulations, unless indicated, were performed while maximizing for the metabolic demand reaction. The proposed metabolic demand reaction represents a drain for specific biomass forming metabolites, all of which are require to be produced optimally.

Table C: Stage-specific bounds and simulated optimal rates of external metabolite exchanges

Exchange name	Promastigote		Amastigote	
	**Lower bound**	**Optimal uptake rate***	**Lower bound**	**Optimal uptake rate***
EX_o2(e)	-1000	-232.14	-65	-65
EX_glu-L(e)	-1000	0	-3	-3
EX_b-D-glucose(e)	-1000	-5.125	-0.2	-0.2
EX_asp-L(e)	-1000	-479.29	-3	-3
EX_pro-L(e)	0	0	-1000	-9.32
EX_hdca	-1000	0	-1000	-0.318
EX_ala-L(e)	-1000	137.75	-2	-2

*calculated with respect to given exchange constraints, while maximizing metabolic demand. Upper bounds of given exchanges were constrained to zero except alanine, which is also produced as an overflow metabolite. Bounds of other internal reactions were constrained between -1000 (lower bound) and 1000 (upper bound) depending upon their reversibility.

The default medium considered for simulating the promastigote and the amastigote scenarios contains glucose, amino sugars, mannose, amino acids (both essential and non-essential), fatty acids, purines and pyrimidines like adenine, guanine, cytosine, uracil, hypoxanthine, inosine, deoxynucleotides, ions, vitamins, CO_2, H_2O, H_2S, HCO_3, NH_3, etc. This media scenario was created with respect to the evidence reported in *Leishmania* literature[12]. The differences in promastigote and amastigote metabolic scenarios arise only due to reduction in the aforementioned uptake rates and hypoxic conditions observed in amastigotes.

5. Fates of carbon sources within the network

The fate of carbohydrates, like glucose (Glc) and mannose (Man), amino sugars like N-acetylglucosamine (Acgam), non-essential amino acids, like alanine (Ala), aspartate (Asp), glutamate (Glu), and hexadecanoate fatty acids (Hdca) was specifically studied in order to compare the model predictions with observations from experimental ^{13}C-isotope labeling profiles of the above metabolites generated in different developmental stages of *L. mexicana*[10]. The *in-silico* stoichiometric-based utilization of each metabolite produced from previous reaction into subsequent reaction/s within the iAS556 network was traced by the contribution of previous reaction flux into its subsequent reaction flux. The ^{13}C-isotope labeling profiles were compared with the predicted steady state reaction fluxes in both the promastigote and amastigote scenarios (Figure S2). A high correlation between the ^{13}C isotope enrichment values of metabolites under uptake of ^{13}C labeled glucose observed in *L. mexicana*[5] and the corresponding predicted reaction steady state fluxes were observed both in intracellular glucose-sufficient promastigote (ρ =

0.788, $P < 10^{-5}$) and glucose-deficient, hypoxic amastigote ($\rho = 0.747$, $P < 10^{-5}$) states, respectively (Figure S2). In both cases, it can be observed that promastigote demonstrates a higher utilization of glucose as compared to amastigote. Few reactions, like myo-inositol-1-phosphate synthase (INO1), alanine aminotransferase (ALATA_Lm), mannan synthesis (MannanSys), display higher steady state fluxes in amastigote as compared to promastigote. The comparison indicates that the metabolites synthesized under glucose input are similar between the predictions and experiments.

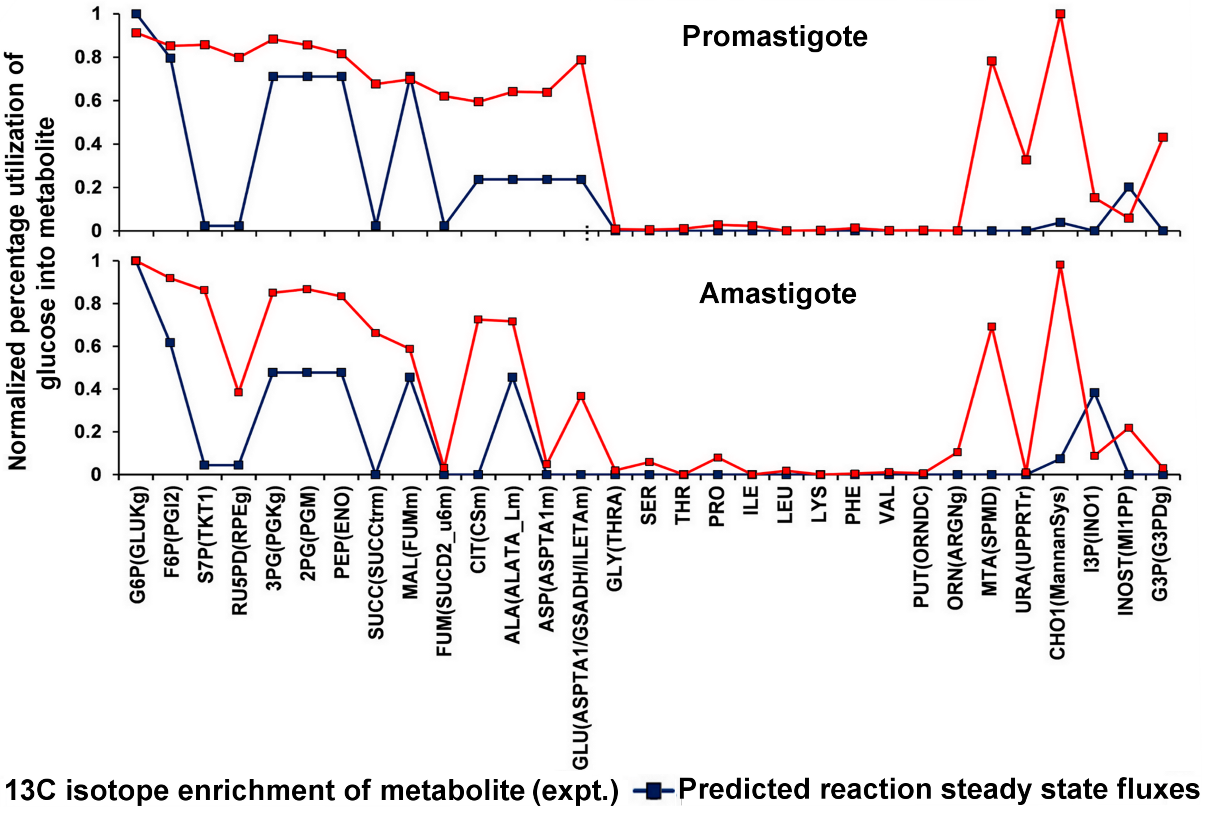

Figure S2. Comparison of predictions with ^{13}C isotope enrichment data – A) Promastigote, B) Amastigote. Red colored lines indicate the flux profiles indicate the available ^{13}C isotope enrichment data in *L. mexicana* (indicated in mol percent, normalized to glucose-6-phosphate) and blue colored lines indicate the reaction fluxes (normalized to hexokinase/glucose uptake) predicted from our model.

6. Reaction knockout analysis

To simulate the effect of the single reaction deletions, the upper and lower bounds of all the reactions were constrained to be zero, sequentially one reaction at a time, and FBA was performed subsequent to each reaction knockout in an *in-silico* rich medium containing glucose; all essential amino acids; non-essential amino acids like cysteine, glycine, aspartate, alanine, glutamine and glutamate; nucleobases like adenine, guanine, hypoxanthine, xanthine, uracil; hexadecanoate, phosphatidic acid, pantothenate and protoheme. Two separate transport reactions of glucose each considering the anomeric, interconvertible forms of glucose are present within the network. In order to avoid redundant glucose uptake from both these transports, one of them was blocked while performing reaction knockouts. A reaction was considered to be essential (lethal phenotype) when biomass reaction flux became zero after applying the aforementioned constraints; else it was deemed to be non-essential (non-lethal phenotype). Lethality predictions remain the same for the model-presumed promastigote and amastigote scenarios, as these scenarios were re-created based only upon stage-specific environmental conditions experienced by the developmental stages of the parasite while maximizing for the same metabolic demand. 188 reactions out of the total 1260 reactions were predicted to be lethal, under the above mentioned model constraints.

Comparison of reaction knockout with experiments

Results from diverse experimental techniques that involved targeted gene deletion, and inhibition of reactions by small molecule analogues were considered for validation purpose. Experimentally determined growth phenotype information for only a few knockouts (43 knockout phenotypes) is explicitly available for any *Leishmania* species (Table E). In most cases (32 out of 43), knockout phenotypes are known and remain the same across both the stages (both-stage knockout phenotypes). Out of the 32, 20 phenotypes were lethal and 12 were non-lethal in both stages. For 11 reactions (one-stage knockout phenotypes), information is either available for promastigote and amastigote stages (10 phenotypes were lethal only in amastigote scenario and 1 phenotype was lethal only in promastigote). Considering the total number of known promastigote knockout phenotypes, 81% phenotypes were accurately predicted from the model. For the known knockout phenotypes of amastigotes, 84% phenotypes were accurately reproduced from the model. Percentage predicted wild type growth rates (standardized to maximum growth in a particular stage) remain the same between the promastigote and the

amastigote situations; except for mitochondrial aconitase, which is non-lethal to the parasite but reduces parasite growth significantly (growth rate reduced by 77%) consistent with experiments[10].

The model predictions can be classified as:

a) Experimentally lethal/non-lethal in both stages and *in-silico* lethal/non-lethal (Perfect predictions): Our model was able to predict the experimentally known phenotypes for the 30 of the 32 both-stage knockout phenotypes perfectly. These knockouts demonstrate either lethal or non-lethal phenotypes from both experiments and predictions.

b) Experimentally non-lethal but lethal *in silico*: only adenine phosphoribosyltransferase (ADPTr) was predicted to be lethal *in silico* as compared to the experimentally known non-lethal phenotype in both the stages (observed in *L. donovani*). ADPTr is experimentally known to provide PRPP[14,15]. The reason for ADPTr becoming lethal in the model is due to the provision of phosphoribosyl pyrophosphate (prpp) to cytosolic phosphoribosylpyrophosphate synthetase (PRPPSic), which utilizes ribose-5-phosphate produced from pentose-phosphate shunt to form AMP (a very essential route). This might be a unique *L. infantum* JPCM5 specific feature. Rest of the inconsistencies is for the stage-specific knockout phenotypes, where the model was able to predict lethality for only either of the stages appropriately.

c) Experimentally lethal but non-lethal *in silico*: Only methylenetetrahydrofolate dehydrogenase (DH1), was predicted to be non-lethal *in silico* as compared to the experimentally lethal phenotype known in both the stages. The main aim of methylenetetrahydrofolate dehydrogenase (DH1) is to produce glycine from serine through dihydrofolate metabolism. According to model predictions, the network structure restricts the essential glycine requirement only through direct uptake. Hence, there is no effect of this pathway on biomass at all. Hence, DH1 and the other reactions of the dihydrofolate pathway (DHFR, TS) remain non-essential. Experimentally, the enzymes of dihydrofolate pathway are essential in nature[22], thereby supporting our prediction. Rest

of the inconsistencies is for the stage-specific knockout phenotypes, where the model was able to predict lethality for only either of the stages appropriately.

For the one-stage knockout phenotypes, no information is available for the other stage. Also, one of the other reasons for the relatively inconsistent predictions is that the same objective function was used for optimization in the promastigote and amastigote states, which is a limitation in our model knockout analysis strategy. As sufficient data for the amastigote biomass requirements is unavailable, separate objective functions for promastigote and amastigote could not be defined. Once these become available, model predictions can be better compared with real phenotypes.

Table E: Comparison of predicted reaction knockout phenotypes with experimentally determined phenotypes

**Reaction (Gene) Name**	**Abbreviation**	**iAS556**	**Expt**	**Organism**	**Reference**	**Stages**	**Predicted wild type growth (%)**	
							**P**	**A**
aconitase	ACONTm	NL	NL	*L. mexicana*	[10]	Both	100%	23%
adenosine deaminase	ADA, ADAer, ADAg, ADAm	NL	NL	*L. donovani*	[13]	Both	100%	100%
adenosine kinase	ADNK1c	NL	NL	*L. donovani*	[13]	Both	100%	100%
1-(5-Phosphoribosyl)-5-amino-4-imidazolecarboxamide:pyrophosphate phosphoribosyltransferase	ADPT2	NL	NL	*L. donovani*	[14,15]	Both	100%	100%
adenine phosphoribosyltransferase	ADPTr	L	NL	*L. donovani*	[14,15]	Both	0%	0%
arginase	ARGNg	NL	NLa	*L. donovani*	[16]	Amastigote	100%	100%
argininosuccinate synthase	ARGSSx	L	La	*L. donovani*	[17]	Amastigote	0%	0%
L-Arginine transport	ARGt	L	L	*L. donovani*	[18]	Both	0%	0%
ATP synthase	ATPSmm	L	L	*L. donovani and L. pifanoi*	[19]	Both	0%	0%
cytochrome-c-oxidase	CYOO6mm	L	L	*L. donovani and L. pifanoi*	[19]	Both	0%	0%
methylenetetrahydrofolate dehydrogenase	DH1	NL	L	*L. major*	[20]	Both	100%	100%
dihydroxyacetonephosphate acyltransferase	DHAPAx_LI	NL	La	*L. major*	[21]	Amastigote	100%	100%
dihydrofolate reductase-thymidine synthase	DHFRc, DHFRm, TS, TSm	NL	NL	*L. major*	[22]	Both	100%	100%
fructose-1,6-bisphosphatase	FBPg	NL	NL	*L. major*	[23]	Both	100%	100%

glucose transporter	GLCBt2	L	La	*L. mexicana*	[21]	Amastigote	0%	0%
glutamylcysteine ligase	GLUCYSL	L	L	*L. infantum*	[24]	Both	0%	0%
N-acetylglucosamine-6-phosphate deacetylase	GNAD	NL	NL	*L. major*	[25]	Both	100%	100%
glucose-6-phosphate N-acetyltransferase	GPAT	NL	NL	*L. major*	[26]	Both	100%	100%
guanine phosphoribosyltransferase	GPRTg	NL	NL	*L. donovani*	[14,15]	Both	100%	100%
glutathionylspermidine synthase	GSS	NL	NL	*L. infantum*	[27]	Both	100%	100%
hypoxanthine phosphoribosyltransferase (hypoxanthine)	HXPRTg	NL	NL	*L. donovani*	[14,15]	Both	100%	100%
myo-inositol-3-phosphate synthase	INO1	NL	La	*L. mexicana*	[21]	Amastigote	100%	100%
trypanothione-dependent glyoxalase I	LGTHL1, LGTHL1m	NL	NL	*L. donovani*	[28]	Both	100%	100%
LIT iron transporter	LIT	L	L	*L. amazonensis*	[29]	Both	0%	0%
mannose-1-phosphate guanylyltransferase	MAN1PT1	L	La	*L. mexicana*	[30]	Amastigote	0%	0%
mannose-6-phosphate isomerase	MAN6PI	L	La	*L. mexicana*	[31,32]	Amastigote	0%	0%
myo-Inositol-1-phosphate synthase	MI1PSB	NL	NL	*L. mexicana*	[33]	Both	100%	100%
5,10-methylenetetrahydrofolate reductase (NADP)	MTHFR1	NL	NL	*L. major*	[34]	Both	100%	100%
NAD-dependent SIR2	NDSIR2	NL	La	*L. infantum*	[35]	Amastigote	100%	100%
ornithine decarboxylase	ORNDC	NL	La	*L. donovani*	[36]	Both	100%	100%
phenylalanine-4-monooxygenase	PHE4MOi	NL	NL	*L. major*	[37]	Both	100%	100%
phosphomannomutase	PMANM	L	La	*L. mexicana*	[32]	Amastigote	0%	0%
pteridine reductase 1	PTR1	NL	NL	*L. major*	[22]	Both	100%	100%
serine-C-palmitoyltransferase	SERPTr	NL	NL	*L. major*	[38]	Both	100%	100%
serine hydroxymethyltranferase (SHMT-S)	SHMTc2	NL	NL	*L. major*	[39]	Both	100%	100%
spermidine synthase	SPMS	L	L	*L. donovani*	[36]	Both	0%	0%
squalene epoxidase	SQLMer	L	L	*L. amazonensis*	[40,41]	Both	0%	0%
squalene synthase	SQSr, SQSm	L	L	*L. chagasi*	[42]	Both	0%	0%
sterol 14-demethylase	ST14DMr	L	L	*L.donovani*	[43]	Both	0%	0%
trypanothione reductase	TRYR	NL	La	*L. donovani*	[44]	Amastigote	100%	100%
trypanothione synthetase	TRYS	L	L	*L. infantum*	[27]	Both	0%	0%
UDP sugar pyrophosphorylase	USPx	NL	NL	*L. major*	[45]	Both	100%	100%
xanthine phosphoribosyltransferase	XPRTgr	NL	NL	*L. donovani*	[14,15]	Both	100%	100%

Both: Promastigote & Amastigote; P: Promastigote; A: Amastigote

L – Lethal, NL – Non-lethal, La- Lethal in Amastigote, NLa – Non-lethal in Amastigote,

% Predicted wild type growth – predicted percentage of maximum growth after a single gene deletion

7. Role of subcellular compartmentalization

The glycosome and mitochondrion are primarily responsible in coercing dependence between enzymes of crucial pathways and the non-essential amino acid motif. To study their roles, both were removed independently and the flux profiles in this scenario were calculated and compared with the normal flux profiles where all the compartments are present (Supplementary Tables S2 and S3). While doing so, the duplicate reactions that would form after removal of subcellular compartment due to dual localizations of enzymes were made singular to eliminate redundancy.

Role of glycosome

To investigate the role of glycosome, model based situations signifying the presence or absence of the glycosome were created. Simulations were performed in both the model-presumed promastigote and amastigote conditions (Figure 3A in Main article). In the promastigote, the presence of glycosome confines the reactions of glycolysis, glycosomal succinate fermentation, and fatty acid β-oxidation thereby limiting their pairing with cytoplasmic reactions for reducing equivalents and ATP. This eventually regulates flux through glycolysis and pentose phosphate pathway. This also promotes drain of glucose via C4 dicarboxylic acid shuttling into mitochondrial TCA while providing reducing equivalents for the non-essential amino acid motif. The non-oxidative arm of the pentose-phosphate pathway along with cytoplasmic glutamate dehydrogenase form reduced NADP (NADPH) used towards formation of glutamate semialdehyde, a primary intermediate for proline biosynthesis. The rest of the reduced NADP produced from glutamate dehydrogenase is utilized by the ultimate step of proline biosynthesis pathway. Similarly, in the amastigote (Figure 3A in Main article), which is characterized by its reduction in glucose uptake, glycosome ensures the utilization of glucose towards mannan via fructose-6-phosphate, myo-inositol phosphate via glucose-6-phosphate, and synthesis of purines (AMP) via oxidative arm of pentose phosphate pathway, thereby reducing the drain of glucose towards utilization within the non-essential amino acid motif. The reduction in glycolytic flux leads to a probable deficiency in availability of NADH for glycosomal succinate fermentation which induces the utilization of fatty acids to compensate for NADH availability in the glycosome. The coupling between cytoplasmic glutamate dehydrogenase and proline biosynthesis is retained, but the reduced availability of glutamate for satisfying cellular demand and cytoplasmic NADPH in absence of the non-oxidative arm of PPP is compensated by proline

uptake and utilization into mitochondria. Hence, in proline deficient conditions, a knockout of cytoplasmic glutamate dehydrogenase produces a lethal phenotype.

The absence of glycosome in the model-presumed promastigote scenario (Figure 3B in Main article) leads to deficiency of AMP production, which is compensated by the drain of glucose into non-oxidative arm of pentose phosphate pathway. This relieves the dependence of lower part of glycolysis on glucose produced from the upper part. Instead, fatty acids replace glucose to provide fructose-6-phosphate which is utilized to form C4 dicarboxylic acids via succinate fermentation to form glutamate from mitochondrial aspartate transaminase. NADH redox coupling between fatty acid β-oxidation, glyceraldehyde-3-phosphate dehydrogenase and succinate fermentation can also be observed. A futile cycle is formed between amino sugar catabolic pathway and pentose phosphate pathway, where fatty acids catabolize to form acetyl-coA, which along with amino sugars enters the cycle to form fructose-6-phosphate. This leads to production of reduced equivalents of NADP via the non-oxidative arm of pentose phosphate pathway and glutamate dehydrogenase which are then utilized for proline biosynthesis to meet the metabolic demand. The remaining reduced equivalents are oxidized to NADP via trypanothione reductase. In the amastigote scenario (Figure 3B in Main article), glycolytic flux is maintained by glucose and fatty acid uptake is totally inhibited. This is contradictory to the real amastigote scenario. The requirement of glutamate within the biomass increases, which is met by an external uptake of proline and its catabolism. The NADP requirements for this purpose are solely fulfilled by trypanothione reductase. Testing under the null hypothesis that the removal of glycosome has no effect on the generated flux profiles; under glucose-deficient conditions, absence of glycosome gives rise to a significantly different flux profile ($P < 0.05$) (Figure S3.B); although under glucose-sufficient conditions (Figure S3.A), the generated profiles seem to be similar thereby supporting the null hypothesis.

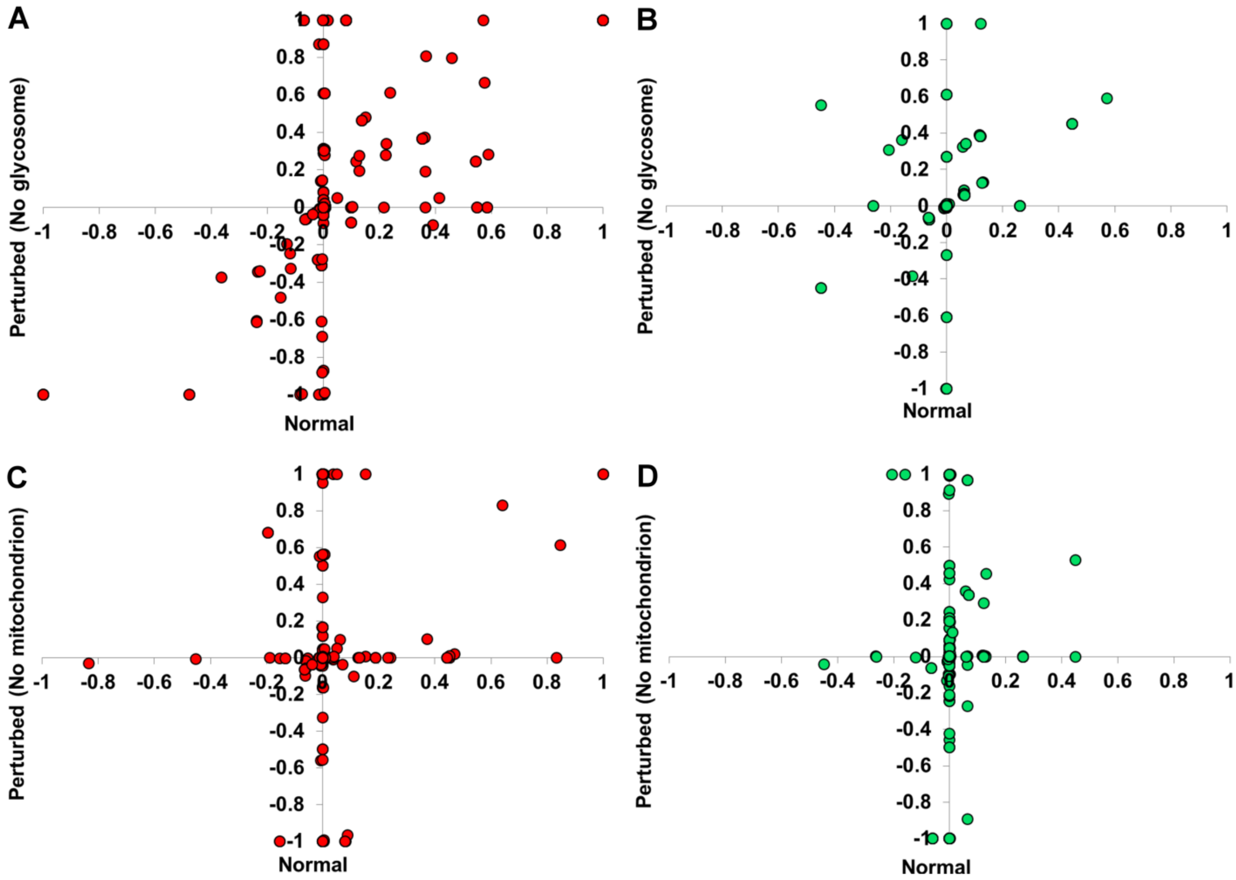

Figure S3 - Comparison of normalized flux profiles of normal and perturbed scenarios with respect to removal of subcellular compartments – A) Glycosome vs. No glycosome, Promastigote (*P* = 0.289, Wilcoxon rank-sum test); B) Glycosome vs. No glycosome, Amastigote (*P* = 0.0317, Wilcoxon rank-sum test); C) Mitochondrion vs. No mitochondrion, Promastigote (*P* = 0.6746, Wilcoxon rank-sum test); D) Mitochondrion vs. No mitochondrion, Amastigote (*P* = 0.0094, Wilcoxon rank-sum test).

Role of mitochondrion

Similar to the glycosome, the mitochondrion was removed from the model and its role was investigated in model-presumed promastigote and amastigote scenarios (Figure 4A in Main article). In the promastigote, the mitochondrion reinforces coupling between glutamate dehydrogenase and proline biosynthesis for NADP redox, and between cytoplasmic malate dehydrogenase and glutamate formation via tyrosine catabolism for NAD redox in the cytoplasm. Glucose catabolism into C4 dicarboxylic acids is utilized into the TCA cycle which is coupled to oxidative phosphorylation via mitochondrial NAD/NADP redox. Glutamate semialdehyde formed from tyrosine catabolism is NAD coupled to mitochondrial glutamate and

pyruvate dehydrogenase. Leucine is utilized to produce intermediates for sterol, heme biosynthesis and glutamate formation, thereby generating reduced FAD that transfer protons for fumarate reduction.

Absence of mitochondrion (Figure 4B in Main article), decouples tyrosine catabolism with proline biosynthesis as the requirements for NADPH production are solely met by glutamate dehydrogenase while coupling with folate degradation and proline biosynthetic pathways. Further, alanine is degraded to form intermediates required for proline synthesis in promastigote and glutamate synthesis in amastigote. A drain for succinate production is activated and excess malate is released into the environment. This impairs the coupling of TCA with oxidative phosphorylation for NAD and NADP regeneration, deeming the TCA cycle to be inactive. The NAD coupling in this case is totally met by the cytoplasmic counterpart of glycolysis and fatty acid biosynthesis. Requirement for asparagine increases and fatty acid uptake from the environment is entirely blocked. This is because the role of pyruvate dehydrogenase complex is completely lost. Testing under the null hypothesis that the removal of mitochondrion has no effect on the generated flux profiles; under glucose-deficient conditions, absence of mitochondrion gives rise to a significantly different flux profile ($P < 0.05$) (Figure S3.D); although under glucose-sufficient conditions (Figure S3.C), the generated profiles seem to be similar thereby supporting the null hypothesis.

Model predictions indicate that the removal of both glycosome and mitochondria does not lead to cessation of growth, suggesting that the important roles of these compartments are only to ensure environmental condition dependent flux coupling between specific pathways.

8. Flux coupling analysis (FCA)

To identify genome-wide flux-coupling (physiological) relationships within the *L. infantum* metabolic network, flux coupling analysis[46] was applied. Flux coupling analysis is a constraint-based procedure that utilizes the stoichiometric and reversibility information of reactions within the metabolic network to identify flux dependencies between a pair of reactions given specific environmental (exchange) constraints. If a non-zero flux in one reaction implies a non-zero flux in the other reaction then the two reactions are coupled with each other. FCA allows the computation of all possible set of coupled reaction subsets given the constraints and does not

depend on a single hypothetical objective function like, FBA, which induces the artificial coupling of network reactions that can optimize the objective. FCA was performed using the F2C2 tool[47] that classifies reactions into 5 categories as per standard definitions of coupled fluxes between a pair of reactions[46];

a) fully coupled pairs (FC) – if a non-zero flux through first reaction induces a non-zero fixed flux through the second reaction and vice versa, then the two reactions form a fully coupled pair,
b) directionally coupled pairs (DC) – if a non-zero flux through the first reaction implies a non-zero flux in the second reaction but the reverse is not possible, then the reactions are directionally coupled to each other,
c) partially coupled pairs (PC) – it is a special case of fully coupled pairs where a non-zero flux dependence exists between the two reactions, the corresponding flux being variable,
d) blocked reactions – if a reaction carries zero flux irrespective of a flux change in any other reaction within the network, it is classified to be blocked,
e) uncoupled reactions – if a reaction pair does not fall into any of the above categories, it was classified as uncoupled.

To avoid hypothetical coupling of large set of reaction fluxes with the biomass reaction, the biomass reaction was removed from the network. Separate, independent reversible drains for metabolites within the biomass were provided in the network allowing for both their uptake and release from/into the environment and then flux-coupling analysis was performed. With this addition, a revised network consisting of 1288 reactions and 1160 metabolites was subjected to flux coupling analysis. FCA within the *L. infantum* iAS556 metabolic network identified 2243 fully coupled pairs, 4 partially coupled pairs, 2460 directionally coupled pairs, 128 uncoupled reactions and 339 blocked reactions. The number of identified blocked reactions within the iAS556 network was also found to be comparable with the *L. major* iAC560[4] and *E. coli* iJO1366[48] metabolic networks. Around 33.48% [865 of 2583] of total reactions within the *E .coli* network, 35.7% [398 of 1112] within the *L. major* network and 26.31% [339 of 1288] within the *L. infantum* iAS556 network were identified to be blocked. We presume that with more availability of information related to *Leishmania* metabolism, the number of blocked reactions would reduce.

The reaction pairs that are coupled can be identified using the flux coupling table computed by the F2C2 tool. The above flux coupled pairs was represented as a mixed flux-coupled graph (Figure 5 of the Main Article) containing both directed and undirected edges that indicates the type of coupling between any two reactions (nodes in the flux coupled graph)[49]. The flux-coupled graph represents a subnetwork (subgraph) of the unipartite reaction projection of the bipartite metabolic network. F2C2 identified 949 unblocked reactions within the *L. infantum* iAS556 metabolic network. The flux-coupled graph was generated using the 949 x 949 adjacency matrix (obtained from the flux coupling table) that contains information of each pair of reactions to be either coupled (1) or uncoupled (0). Fully and partially coupled reaction pairs are reversibly coupled and hence within the flux-coupled graph, were connected by a single undirected edge. Directionally coupled reaction pairs were connected by a single directed edge from one node to another. Isolated nodes with zero connectivity represent the completely uncoupled reactions. With this graph representation, it is possible to identify the total number of reactions to which a given reaction can be coupled by computing the degree centrality of each node of the flux-coupled graph. Local clustering coefficient was computed for each node to predict the probability that a particular reaction would be a part of a strongly connected motif within the network. The F2C2 tool is available as a set of MATLAB subroutines. Also, topological analysis for the flux-coupled graph was performed using subroutines developed in-house and implemented in MATLAB version R2012a. Gephi version 0.8.2 was used to visualize the flux-coupled graph.

9. Perturbation of physiological coupling relationships

The network was perturbed by simultaneously removing a series of up to 20 out of the 647 known gene-associated reactions randomly from the metabolic network, while re-calculating the flux relationships within the perturbed network after each deletion set. 1000 random simulations were performed for each case (Figure S4). Each deletion biologically represents the complete loss of function of a given gene or sets of genes that absolutely inhibit their corresponding reactions. The median number of flux-coupled pairs [both fully (FC) and directionally coupled (DC)] almost remains the same up to 5 simultaneous deletions (< 1% decrease in DC and < 0.2% decrease in FC pairs), after which the numbers gradually decrease with the largest change observed for 20 random deletions (25.6% decrease in DC and 10.7% decrease in FC pairs).

Median number of reactions represented within strongly connected motifs (local clustering coefficient = 1) remains invariably constant across reaction deletions. As weakly connected motif (0 < local clustering coefficient < 1) or totally non-motif reactions (local clustering coefficient = 0) are high in number, chances of deletions to occur in strongly connected motifs becomes drastically low and hence, their number remain largely invariable.

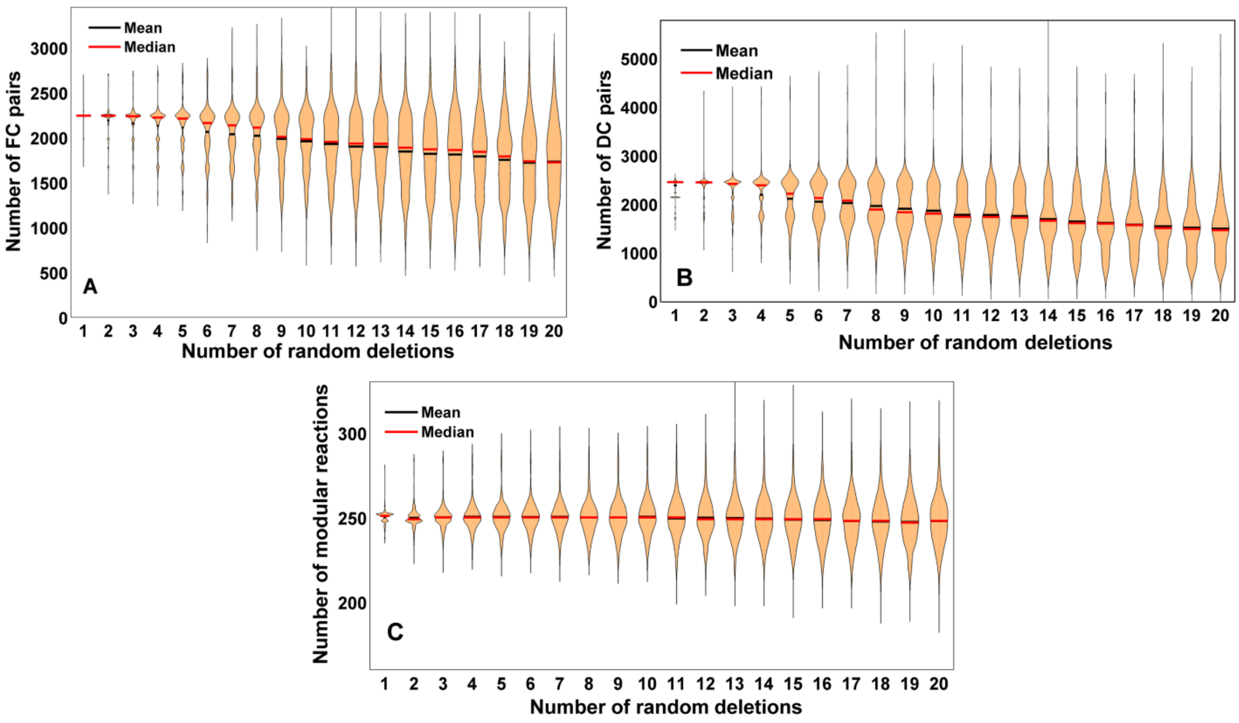

Figure S4 - Effect of random deletions on physiological flux coupling relationships. Violin plot indicating - A) Number of FC pairs, B) Number of DC pairs, C) Number of modular reactions. A total of 1000 random sets of deletions were performed for each case.

10. Additional Files (Legends)

Supplementary Data S1 – List of genes, proteins, reactions, subcellular locations, pathways and other essential information of *Leishmania infantum* JPCM5 metabolism used for iAS556 model reconstruction.

Supplementary Data S2 - The COBRA model structure of the *L. infantum* iAS556 genome-scale metabolic network in '.mat' format.

Supplementary Table S1 - Fates of different external metabolites expressed in the form of reaction fluxes generated for both the promastigote and amastigote scenarios.

Supplementary Table S2 - Flux profiles predicted for the network reactions for the normal and perturbed (no glycosome) scenarios.

Supplementary Table S3 - Flux profiles predicted for the network reactions for the normal and perturbed (no mitochondrion) scenarios.

Supplementary Table S4 - Total number of flux couplings representing the sum of directional, full and partial couplings of a particular reaction after performing flux coupling analysis and the corresponding coupled component number in which a particular reaction is involved.

Supplementary Table S5 - Total number of coupled components in which each biomass metabolite is involved and its corresponding pathway.

Supplementary References

1. Subramanian, A., Jhawar, J. & Sarkar, R. R. Dissecting *Leishmania infantum* energy metabolism - A systems perspective. *PLoS One* **10**, (2015).

2. Silva, A. M., Cordeiro-da-Silva, A. & Coombs, G. H. Metabolic variation during development in culture of *Leishmania donovani* promastigotes. *PLoS Negl Trop Dis* **5**, e1451 (2011).

3. Thorleifsson, S. G. & Thiele, I. rBioNet: A COBRA toolbox extension for reconstructing high-quality biochemical networks. *Bioinformatics* **27**, 2009–2010 (2011).

4. Chavali, A. K., Whittemore, J. D., Eddy, J. A., Williams, K. T. & Papin, J. A. Systems analysis of metabolism in the pathogenic trypanosomatid *Leishmania major*. *Mol. Syst. Biol.* **4**, 177 (2008).

5. Jamdhade, M. D. et al. Comprehensive proteomics analysis of glycosomes from *Leishmania donovani*. *Omics* **19**, 157–170 (2015).

6. Zhang, K. & Beverley, S. M. Phospholipid and sphingolipid metabolism in *Leishmania*. *Mol. Biochem. Parasitol.* **170**, 55–64 (2010).

7. Roberts, S. B. et al. Proteomic and network analysis characterize stage-specific metabolism in *Trypanosoma cruzi*. *BMC Syst. Biol.* **3**, 1 (2009).

8. Orth, J. D., Thiele, I. & Palsson, B. Ø. What is flux balance analysis? *Nat. Biotechnol.* **28**, 245–8 (2010).

9. Schellenberger, J. et al. Quantitative prediction of cellular metabolism with constraint-based models: the COBRA Toolbox v2. 0. *Nat. Protoc.* **6**, 1290–1307 (2011).

10. Saunders, E. C. et al. Induction of a stringent metabolic response in intracellular stages of *Leishmania mexicana* leads to increased dependence on mitochondrial metabolism. *PLoS Pathog.* **10**, e1003888 (2014).

11. Ravikrishnan, A. & Raman, K. Critical assessment of genome-scale metabolic networks: the need for a unified standard. Brief. Bioinform. 16, 1057–1068 (2015).

12. McConville, M. J. & Naderer, T. Metabolic pathways required for the intracellular survival of *Leishmania*. *Annu. Rev. Microbiol.* **6**, 543–561 (2011).

13. Boitz, J. M., Strasser, R., Hartman, C. U., Jardim, A. & Ullman, B. Adenine aminohydrolase from *Leishmania donovani* unique enzyme in parasite purine metabolism. *J. Biol. Chem.* **287**, 7626–7639 (2012).

14. Carter, N. S., Yates, P., Arendt, C. S., Boitz, J. M. & Ullman, B. in Drug Targets in Kinetoplastid Parasites 141–154 (Springer, 2008).

15. Boitz, J. M. & Ullman, B. *Leishmania donovani* singly deficient in HGPRT, APRT or XPRT are viable *in vitro* and within mammalian macrophages. *Mol. Biochem. Parasitol.* **148**, 24–30 (2006).

16. Boitz, J. M. et al. Arginase is essential for survival of *Leishmania donovani* promastigotes but not intracellular amastigotes. *Infect. Immun.* **IAI--00554** (2016).

17. Lakhal-Naouar, I. et al. *Leishmania donovani* argininosuccinate synthase is an active enzyme associated with parasite pathogenesis. *PLOS Negl Trop Dis* **6**, e1849 (2012).

18. Shaked-Mishan, P. et al. A novel high-affinity arginine transporter from the human parasitic protozoan *Leishmania donovani*. *Mol. Microbiol.* **60**, 30–38 (2006).

19. Luque-Ortega, J. R., van't Hof, W., Veerman, E. C. I., Saugar, J. M. & Rivas, L. Human antimicrobial peptide histatin 5 is a cell-penetrating peptide targeting mitochondrial ATP synthesis in *Leishmania*. *FASEB J.* **22**, 1817–28 (2008).

20. Murta, S. M. F., Vickers, T. J., Scott, D. A. & Beverley, S. M. Methylene tetrahydrofolate dehydrogenase/cyclohydrolase and the synthesis of 10-CHO-THF are essential in *Leishmania major*. *Mol. Microbiol.* **71**, 1386–1401 (2009).

21. McConville, M. J., de Souza, D., Saunders, E., Likic, V. A. & Naderer, T. Living in a phagolysosome; metabolism of *Leishmania* amastigotes. *Trends Parasitol.* **23**, 368–375 (2007).

22. Nare, B., Hardy, L. W. & Beverley, S. M. The roles of pteridine reductase 1 and dihydrofolate reductase-thymidylate synthase in pteridine metabolism in the protozoan parasite *Leishmania major*. *J. Biol. Chem.* **272**, 13883–13891 (1997).

23. Naderer, T. et al. Virulence of *Leishmania major* in macrophages and mice requires the gluconeogenic enzyme fructose-1, 6-bisphosphatase. *Proc. Natl. Acad. Sci.* **103**, 5502–5507 (2006).

24. Mukherjee, A., Roy, G., Guimond, C. & Ouellette, M. The γ-glutamylcysteine synthetase gene of *Leishmania* is essential and involved in response to oxidants. *Mol. Microbiol.* **74**, 914–927 (2009).

25. Naderer, T., Heng, J. & McConville, M. J. Evidence that intracellular stages of *Leishmania major* utilize amino sugars as a major carbon source. *PLoS Pathog* **6**, e1001245 (2010).

26. Naderer, T. et al. Intracellular survival of *Leishmania major* depends on uptake and degradation of extracellular matrix glycosaminoglycans by macrophages. *PLoS Pathog* **11**, e1005136 (2015).

27. Sousa, A. F. et al. Genetic and chemical analyses reveal that trypanothione synthetase but not glutathionylspermidine synthetase is essential for *Leishmania infantum*. *Free Radic. Biol. Med.* **73**, 229–238 (2014).

28. Chauhan, S. C. & Madhubala, R. Glyoxalase I gene deletion mutants of *Leishmania donovani* exhibit reduced methylglyoxal detoxification. *PLoS One* **4**, e6805 (2009).

29. Jacques, I., Andrews, N. W. & Huynh, C. Functional characterization of LIT1, the *Leishmania amazonensis* ferrous iron transporter. *Mol. Biochem. Parasitol.* **170**, 28–36 (2010).

30. Garami, A. & Ilg, T. Disruption of mannose activation in *Leishmania mexicana*: GDP-mannose pyrophosphorylase is required for virulence, but not for viability. *EMBO J.* **20**, 3657–3666 (2001).

31. Garami, A. & Ilg, T. The role of phosphomannose isomerase in *Leishmania mexicana* glycoconjugate synthesis and virulence. *J. Biol. Chem.* **276**, 6566–6575 (2001).

32. Garami, A., Mehlert, A. & Ilg, T. Glycosylation defects and virulence phenotypes of *Leishmania mexicana* phosphomannomutase and dolicholphosphate-mannose synthase gene deletion mutants. *Mol. Cell. Biol.* **21**, 8168–8183 (2001).

33. Ilg, T. Generation of myo-inositol-auxotrophic *Leishmania mexicana* mutants by targeted replacement of the myo-inositol-1-phosphate synthase gene. *Mol. Biochem. Parasitol.* **120**, 151–156 (2002).

34. Vickers, T. J. et al. Biochemical and genetic analysis of methylenetetrahydrofolate reductase in *Leishmania* metabolism and virulence. *J. Biol. Chem.* **281**, 38150–38158 (2006).

35. Gazanion, E. et al. The *Leishmania* nicotinamidase is essential for NAD+ production and parasite proliferation. *Mol. Microbiol.* **82**, 21–38 (2011).

36. Gilroy, C., Olenyik, T., Roberts, S. C. & Ullman, B. Spermidine synthase is required for virulence of *Leishmania donovani*. *Infect. Immun.* **79**, 2764–2769 (2011).

37. Lye, L.-F., Kang, S. O., Nosanchuk, J. D., Casadevall, A. & Beverley, S. M. Phenylalanine hydroxylase (PAH) from the lower eukaryote *Leishmania major*. *Mol. Biochem. Parasitol.* **175**, 58–67 (2011).

38. Zhang, K. et al. Sphingolipids are essential for differentiation but not growth in *Leishmania*. *EMBO J.* **22**, 6016–6026 (2003).

39. Roy, G. & Ouellette, M. Inactivation of the cytosolic and mitochondrial serine hydroxymethyl transferase genes in *Leishmania major*. *Mol. Biochem. Parasitol.* **204**, 106–110 (2015).

40. Vannier-Santos, M. A., Urbina, J. A., Martiny, A., Neves, A. & Souza, W. Alterations induced by the antifungal compounds ketoconazole and terbinafine in *Leishmania*. *J. Eukaryot. Microbiol.* **42**, 337–346 (1995).

41. Chawla, B. & Madhubala, R. Drug targets in *Leishmania*. *J. Parasit. Dis.* **34**, 1–13 (2010).

42. Granthon, A. C. et al. Alterations on the growth and ultrastructure of *Leishmania chagasi* induced by squalene synthase inhibitors. *Vet. Parasitol.* **146**, 25–34 (2007).

43. McCall, L.-I. et al. Targeting ergosterol biosynthesis in *Leishmania donovani*: essentiality of sterol 14alpha-demethylase. *PLoS Negl Trop Dis* **9**, e0003588 (2015).

44. Tovar, J., Wilkinson, S., Mottram, J. C. & Fairlamb, A. H. Evidence that trypanothione reductase is an essential enzyme in *Leishmania* by targeted replacement of the tryA gene locus. *Mol. Microbiol.* **29**, 653–660 (1998).

45. Lamerz, A.-C. et al. Deletion of UDP-glucose pyrophosphorylase reveals a UDP-glucose independent UDP-galactose salvage pathway in *Leishmania major*. *Glycobiology* **20**, 872–882 (2010).

46. Burgard, A. P., Nikolaev, E. V, Schilling, C. H. & Maranas, C. D. Flux coupling analysis of genome-scale metabolic network reconstructions. *Genome Res.* **14**, 301–312 (2004).

47. Larhlimi, A., David, L., Selbig, J. & Bockmayr, A. F2C2: a fast tool for the computation of flux coupling in genome-scale metabolic networks. *BMC Bioinformatics* **13**, 57 (2012).

48. Orth, J. D. et al. A comprehensive genome-scale reconstruction of *Escherichia coli* metabolism—2011. *Mol. Syst. Biol.* **7**, 535 (2011).

49. Subramanian A, Sarkar R. R. (2016) Network structure and enzymatic evolution in *Leishmania* metabolism: a computational study. *Proceedings of the International Symposium on Mathematical and Computational Biology-BIOMAT 2015. World Scientific*, ISBN: 978-981-3141-90-2, 1 - 20
